# The Deubiquitinase USP4 Stabilizes Twist1 Protein to Promote Lung Cancer Cell Stemness

**DOI:** 10.3390/cancers12061582

**Published:** 2020-06-15

**Authors:** Fengtian Li, Qingyong Hu, Tao He, Jing Xu, Yong Yi, Siyi Xie, Liangping Ding, Mengyuan Fu, Rongtian Guo, Zhi-Xiong Jim Xiao, Mengmeng Niu

**Affiliations:** Center of Growth, Metabolism and Aging, Key Laboratory of Bio-Resource and Eco-Environment, Ministry of Education, College of Life Sciences, Sichuan University, Chengdu 610065, China; lft549544593@live.com (F.L.); huqi163@yeah.net (Q.H.); taohe1987_ws@163.com (T.H.); xuj348@nenu.edu.cn (J.X.); yy-yiyong@scu.edu.cn (Y.Y.); zoexie_xsy@hotmail.com (S.X.); DingLP@hotmail.com (L.D.); mengyuan_fmy@163.com (M.F.); GRT9307@163.com (R.G.)

**Keywords:** USP4, Twist1, lung cancer, CSCs, stemness

## Abstract

Lung cancer stem cells (CSCs) play a pivotal role in tumor development, drug resistance, metastasis and recurrence of lung cancer. Thus, it is of great importance to study the mechanism by which CSCs are regulated. In this study, we demonstrate that the deubiquitinase USP4 is critically important in promoting lung cancer stemness. Silencing of USP4 leads to reduction of Oct4 and Sox2 expression, decreased CD133+ cell population and inhibition of tumorsphere formation. Conversely, ectopic expression of USP4 significantly enhances lung cancer cell stemness, which is effectively rescued by simultaneous silencing of Twist1. Mechanistically, we identified USP4 as a novel deubiquitinase of Twist1. USP4 binds to, deubiquitinates and stabilizes Twist1 protein. Furthermore, we show that USP4 expression is elevated in human lung cancer specimens and is positively correlated with Twist1 expression. High expression of USP4/Twist1 is associated with poor clinical outcomes of lung cancer patients. Together, this study highlights an important role for USP4 in lung cancer stemness and suggests USP4 as a potential target for lung cancer diagnosis and treatment.

## 1. Introduction

Lung cancer is the most common cancer worldwide and a leading cause of cancer-related death [1]. Histopathologically, lung cancer is categorized into small cell lung cancer (SCLC) and non-small cell lung cancer (NSCLC), of which NSCLC accounts for 85% of all lung cancer cases. In recent years, development of NSCLC therapy, including chemotherapy, radiotherapy, targeted therapy and immunotherapy, greatly improves both survival and prognosis for patients [2]. However, a major challenge in NSCLC treatment is that most of NSCLC patients do not respond well to immunotherapy and that tumor recurrence and drug resistance after treatment remain [3]. Thus, it is of great importance to illustrate new mechanisms and new drug targets involved in NSCLC tumorigenesis and drug resistance.

It has been well documented that cancer stem cells (CSCs) play a critical role in cancer development, tumorigenesis, drug resistance, metastasis and recurrence [4,5,6,7]. CSCs are a small sub-population of tumor cells with the capacity for self-renewal, differentiation, tumor initiation and innate resistance to chemotherapy and radiation. These characters associated with CSCs are collectively called stemness. In NSCLC, CSCs can be identified and characterized by expression of CSCs markers, including CD133, CD44 and aldehyde dehydrogenase (ALDH) [8,9,10,11]. Several embryonic transcription factors, including Oct4, Sox2, Nanog, MYC and KLF4, are essential in the regulation of stemness of CSCs [8,12,13,14,15]. In addition, CSCs characteristically exhibit activation of several highly conserved pathways involved in cell renewal and tissue homeostasis, including Hedgehog, Notch, WNT/β-Catenin and TNF-α, to promote cell survival, self-renewal, and metastasis [16,17,18,19]. Targeting proteins critical for CSCs stemness has been a potential strategy for cancer treatment [20,21].

Twist1, a basic helix-loop-helix (bHLH) transcription factor, plays a critical role in promoting EMT, tumor metastasis, cancer stemness and drug resistance [5,6]. Twist1 can form homo-dimers or hetero-dimers with other bHLH proteins, which directly regulate the transcription of EMT or stemness-related genes [22,23]. Twist1 is a key transcription factor to upregulate expression of Oct4 and Sox2 [24,25]. Expression of Twist1 is elevated in various human cancers, including cancers of lung, breast, prostate and liver [26,27,28,29,30,31,32,33]. It has been reported that Twist1 is critical for oncogene-driven cell proliferation since silencing of Twist1 induces senescence in lung adenocarcinoma cells driven by various oncogenic drivers [26]. Activation of TGF-β signaling upregulates Twist1 expression, which in turn promotes lung cancer stemness [34]. Several E3 ubiquitin ligases, including FBXL14, β-TRCP, FBXO45 and Pirh2, have been shown to bind to and degrade Twist1 protein [35,36,37]. In addition, HR23A protein, a poly-ubiquitin chain carrier, targets and degrades Twist1 to inhibit lung cancer stemness [38].

Deubiquitinating enzymes (DUBs) are a group of proteases that catalyze the cleavage of protein–ubiquitin bonds. USPs (ubiquitin-specific proteases), the largest subfamily of DUB, play critical roles in development of various malignant tumors [39,40]. USP4 is the first deubiquitinating enzyme identified in mammalian cells [41] and is frequently overexpressed in various cancers, including lung adenocarcinoma and glioblastoma [42,43]. A body of evidence indicates that USP4 functions as a potent oncogene, through regulating p53 signaling, Wnt/β-catenin and TGF-β/SMAD signaling pathways, to promote tumorigenesis and cancer development [44,45,46,47]. However, whether USP4 directly regulates lung cancer stemness remains unclear.

In this study, we demonstrate that USP4 is critically important in maintaining lung cancer cell stemness. We show that USP4 is a novel deubiquitinating enzyme of Twist1 protein. These results highlight USP4 as a critical factor in promoting lung cancer stemness and as a potentially useful lung cancer prognosis marker.

## 2. Results

### 2.1. USP4 Promotes Lung Cancer Cell Stemness

**Figure 1:**
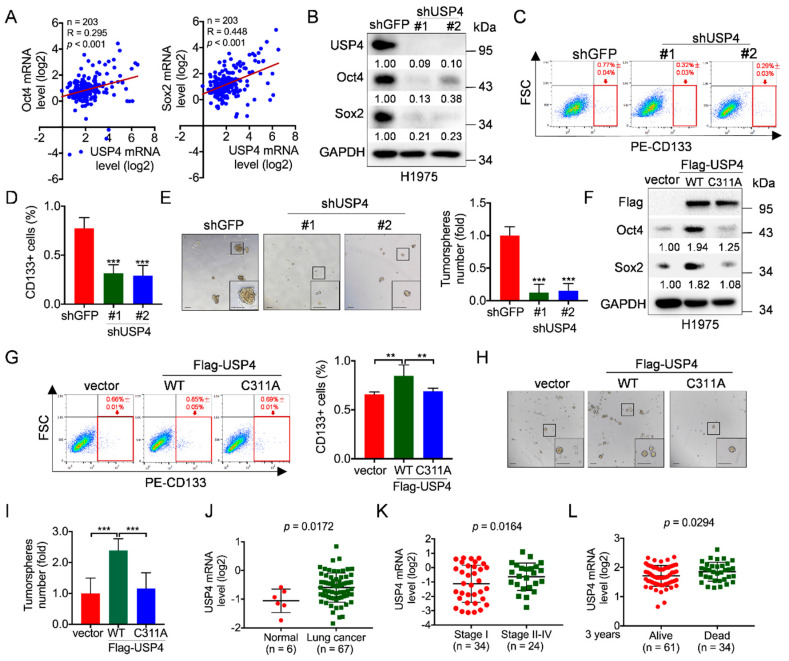
USP4 promotes lung cancer cell stemness and its high expression is correlated with human lung cancer patients. (**A**) The Oncomine dataset “Bhattacharjee Lung” was used to analyze Pearson correlation of USP4 and Oct4/Sox2 expression. (**B**–**E**) H1975 cells stably expressing shRNA against USP4 (shUSP4-#1 or shUSP4-#2) were subjected to (**B**) Western blot analyses, (**C**–**D**) FACS analyses for CD133-stained cells or (**E**) tumorsphere formation assay. Respective images and quantitation were shown. Data from three independent experiments in triplicates were presented as means ± SD. *** *p* < 0.001. Scale bar = 100 μm. (**F**–**I**) H1975 cells stably expressing Flag-USP4 or Flag-USP4^C311A^ were subjected to (**F**) Western blot analyses, (**G**) FACS analyses for CD133-stained cells or (**H**–**I**) tumorsphere formation assay. Respective images and quantitation were shown. Data from three independent experiments in duplicates were presented as means ± SD. ** *p* < 0.01, *** *p* < 0.001. Scale bar = 100 μm. (**J**) The Oncomine dataset “Gaber lung” was used to analyze USP4 mRNA levels in normal human lung tissues and lung cancers. (**K**) The Oncomine dataset “Bild lung” was used to analyze USP4 mRNA levels in stage I or stage II-IV human lung cancers. (**L**) The Oncomine dataset “Raponi lung” was used to analyze USP4 mRNA levels in 3 year-alive or 3 year-dead human lung cancer patients.

Cancer stem cells (CSCs) play a pivotal role in lung cancer development, metastasis and recurrence [4,5,6,7]. Thus, it is of great importance to understand the molecular bases by which CSCs are regulated. Given the critical roles of ubiquitin-specific proteases (USPs) in regulation of protein stability and cancer development, we aimed to identify novel USP(s) in the regulation of CSCs. Therefore, we first analyzed the correlation of expression between USPs and stemness transcript factors exemplified by Oct4/Sox2, utilizing clinical lung cancer samples from Oncomine database [48]. As shown in Figure 1A and Figure S1A–I, expression of USP1 was significantly correlated with expression of Oct4 and Sox2 in human lung cancers, in keeping with previous reports [49,50]. Notably, expression of USP4 was also significantly correlated with expression of Oct4 (R = 0.295; *p* < 0.001; *n* = 203) and Sox2 (R = 0.448; *p* < 0.001; *n* = 203), suggesting that USP4 is a potential positive regulator of lung cancer stemness.

Next, we aimed to confirm the relationship between USP4 and Oct4/Sox2. We used shRNAs specific for USP4 to facilitate the knockdown of USP4 in human non-small cell lung cancer (NSCLC) H1975 and A549 cells. As shown in Figure 1B and Figure S2A, silencing of USP4 significantly reduced Oct4 and Sox2 protein expression, suggesting that silencing of USP4 can inhibit lung cancer cell stemness. Indeed, silencing of USP4 significantly led to reduced population of CD133+ cells, a marker for CSCs in NSCLC, concomitant with reduced tumorsphere formation (Figure 1C–E and Figure S3A–C). Conversely, ectopic expression of wild-type USP4, but not USP4^C311A^ mutant defective in deubiquitinating enzymatic activity, significantly upregulated Oct4 and Sox2 protein expression, concomitant with both increased population of CD133+ cells and increased capacity of tumorsphere formation (Figure 1F–I, Figure S2B and Figure S4A–C). Together, these data indicate that USP4 is a critical factor to promote lung cancer stemness, which is dependent on its deubiquitinating enzymatic activity.

These results prompted us to verify expression levels of USP4 in lung cancer. Clinical analyses of Oncomine “Gaber lung” dataset showed that USP4 mRNA levels were elevated in lung cancer specimens compared to normal tissues (Figure 1J), and analyses of Oncomine “Bild lung” dataset showed that stage II-IV lung cancer specimens had elevated USP4 mRNA levels compared to stage I lung cancer specimens (Figure 1K). Furthermore, higher levels of USP4 mRNA were significantly associated (*p* = 0.0294) with poor overall three-year survival of lung cancer patients (Figure 1L). Together, these findings indicate that USP4 is critically important in promoting lung cancer stemness and is associated with lung cancer clinical prognosis.

### 2.2. USP4 Promotes Lung Cancer Stemness Via Upregulation of Twist1 Protein Expression

**Figure 2:**
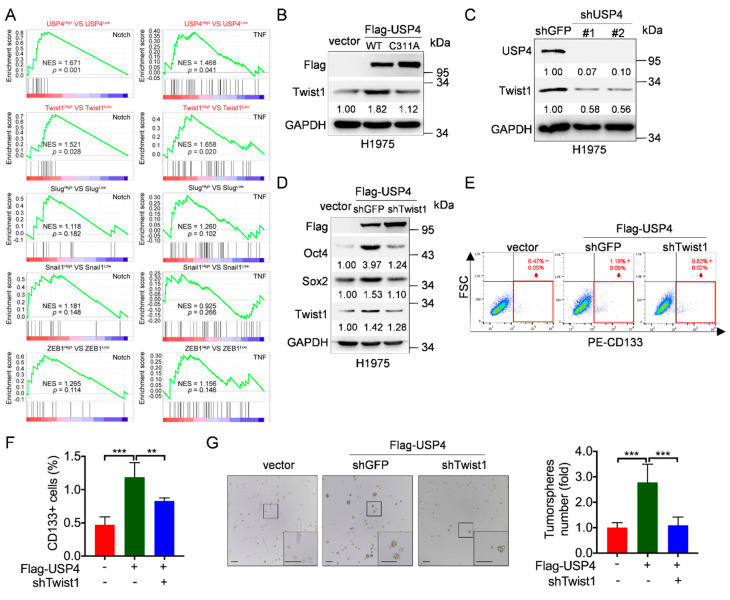
USP4 promotes lung cancer stemness via upregulation of Twist1 protein expression. (**A**) Gene Set Enrichment Analyses (GSEA) analyzed the effects of USP4 or transcriptional factors associated with EMT (Twist1, Slug, Snail and ZEB1) on Notch signaling and TNF signaling. GSE19804 dataset was used to these analyses. NES: normalized enrichment score. (**B**) H1975 cells stably expressing Flag-USP4 or Flag-USP4^C311A^ were subjected to Western blot analyses. (**C**) H1975 cells stably expressing shRNAs against USP4 (shUSP4-#1 or shUSP4-#2) were subjected to Western blot analyses. (**D**–**G**) H1975 cells stably expressing Flag-USP4 and either shTwist1 or shGFP were subjected to (**D**) Western blot analyses, (**E**–**F**) FACS analyses for CD133-stained cells or (**G**) tumorsphere formation assay. Respective images and quantitation were shown. Data from three independent experiments in duplicates were presented as means ± SD. ** *p* < 0.01, *** *p* < 0.001. Scale bar = 100 μm.

We then investigated the molecular bases by which USP4 promotes lung cancer stemness. It has been reported that EMT-associated transcriptional factors, including Twist1, Snail, Slug and ZEB1, play a critical role in lung cancer stemness [34,38]. We therefore performed Gene Set Enrichment Analyses (GSEA) on gene signatures of Notch and TNF signaling, both of which are important in maintaining lung cancer stemness [16,19]. As shown in Figure 2A, high expression of both USP4 and Twist1 was significantly associated with gene signatures of Notch and TNF signaling, suggesting that Twist1 may play a role in USP4-mediated lung cancer stemness. We then examined the effect of USP4 on Twist1 expression. As shown in Figure 2B and Figure S5A, ectopic expression of USP4, but not USP4^C311A^, led to a significant increase in Twist1 protein expression in H1975 cells. Conversely, silencing of USP4 dramatically reduced Twist1 protein expression in H1975 and A549 cells (Figure 2C and Figure S3A). Consistently, ectopic expression of USP4 also increased Twist1 protein expression in A549 cells (Figure S4A).

To investigate the causative role of Twist1 in USP4-mediated lung cancer stemness, we performed the rescuing experiments. As shown in Figure 2D, silencing of Twist1 completely rescued USP4-induced upregulation of Oct4 and Sox2 protein expression. Consistently, silencing of Twist1 significantly reversed USP4-induced increase of CD133+ cell population and tumorsphere formation (Figure 2E–G and Figure S5B). Together, these results demonstrate that Twist1 is a critical downstream effector in USP4-induced lung cancer stemness.

### 2.3. USP4 Is a Deubiquitinase of Twist1 Protein

**Figure 3:**
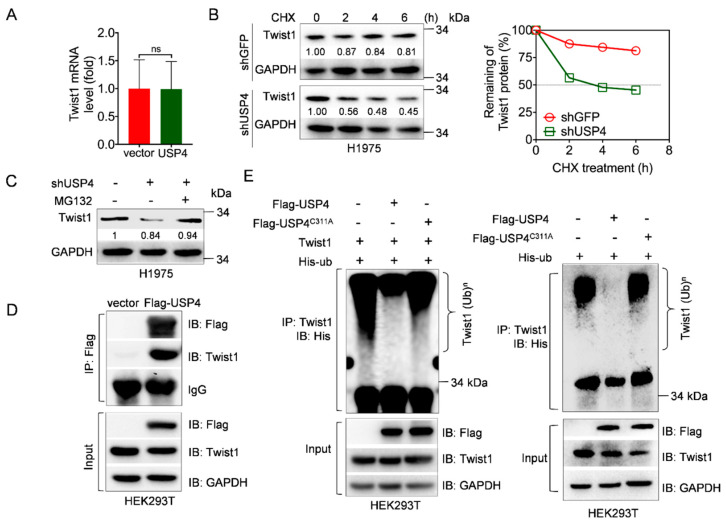
USP4 is a deubiquitinase of Twist1 protein. (**A**) H1975 cells stably expressing USP4 or a vector control were subjected to Q-PCR analyses. ns stands for no significance. (**B**) H1975 cells stably expressing shUSP4 or shGFP were treated with 50 μg/mL cycloheximide (CHX) for an indicated time interval, and then subjected to Western blot analyses. Twist1 protein levels were quantified and the plot was presented. (**C**) H1975 cells stably expressing shRNAs against USP4 (shUSP4) were treated with or without 20 μM MG132 for 12 h prior to Western blot analyses. (**D**) HEK293T cells were transfected with Flag-USP4 expressing plasmids for 36 h. Cells were treated with 20 μM MG132 for 6 h prior to Co-IP experiments. (**E**) HEK293T cells were co-transfected with Twist1 and His-ubiquitin in the presence of either Flag-USP4 or Flag-USP4^C311A^ expressing plasmids for 36 h (left panel). HEK293T cells were co-transfected with His-ubiquitin and either Flag-USP4 or Flag-USP4^C311A^ expressing plasmids for 36 h (right panel). Cells were then treated with 20 μM MG132 for 6 h prior to immunoprecipitation (IP) and Western blot analyses.

We next investigated the mechanism by which USP4 upregulates Twist1 expression. As shown in Figure 3A, ectopic expression of USP4 had little effects on the steady-state Twist1 mRNA levels. Instead, silencing of USP4 significantly shortened the half-life of Twist1 protein (Figure 3B). In addition, treatment with MG132, a well-known proteasome inhibitor, markedly rescued silencing of USP4-induced downregulation of Twist1 (Figure 3C). These results indicate that USP4 stabilizes Twist1 protein via proteasome pathway. 

Next, we investigated whether USP4 can function as deubiquitinating enzyme for Twist1. To this end, we first asked whether USP4 can form a stable protein complex with Twist1. Co-Immunoprecipitation assay showed that USP4 interacted with Twist1 (Figure 3D). Moreover, ectopic expression of USP4, but not USP4^C311A^, dramatically removed polyubiquitin of endogenous Twist1 *in vivo* (Figure 3E). These results indicate that USP4 is a novel deubiquitinating enzyme to protect Twist1 from proteasome-mediated degradation. 

### 2.4. Clinical Validation of Correlation between USP4 and Twist1 Expression and Its Association with Overall Survival in Lung Cancer 

**Figure 4:**
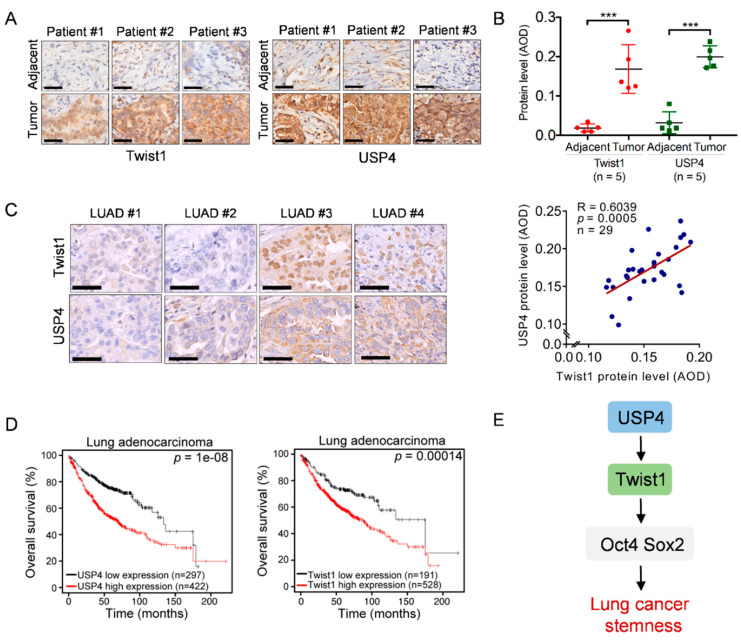
High expression of USP4 is positively correlated with Twist1 and associated with poor overall survival in human lung cancers. (**A**,**B**) Consecutive tissue slides derived from human lung adnocarcinoma (treatment-naïve) and adjacent normal tissues were subjected to immunohistochemistry (IHC) for expression of USP4 and Twist1 expression. Representative images of IHC staining were shown (**A**). Stainings were quantified by average optical density (AOD) (**B**). *** *p* < 0.001. Scale bar = 50 μm. (**C**) Consecutive tissue microarray slides derived from human lung adenocarcinoma (LUAD) were subjected to IHC analyses for Pearson correlation of USP4 and Twist1 expression. Representative images of IHC staining were shown. Stainings were quantified by average optical density (AOD). Scale bar = 50 μm. (**D**) Kaplan–Meier plots of overall survival of human lung cancer patients were stratified by the USP4 or Twist1 mRNA expression levels in the patient tumor samples. (**E**) A working model depicting that USP4 deubiquitinates and stabilizes Twist1 protein to promote lung cancer stemness.

Our data indicate that USP4 stabilizes Twist1 to promote lung cancer cell stemness, which prompted us to verify the clinical relevance of USP4–Twist1 in human lung cancer. As shown in Figure 4A,B, compared to adjacent tissues, expression of both USP4 and Twist1 proteins were high in biopsy specimens of lung adenocarcinoma patients. We further examined the clinical correlation between USP4 and Twist1 using tissue microarrays of lung adenocarcinoma. A positive correlation was observed between USP4 and Twist1 expression (R = 0.6039, *p* = 0.0005) (Figure 4C). Clinical analysis by Kaplan–Meier dataset showed that lung cancer patients with high USP4 or Twist1 levels exhibited poor overall survival (OS) (Figure 4D). Together, these results indicate that the USP4–Twist1 axis plays a critical role in lung cancer stemness and clinical prognoses (Figure 4E).

## 3. Discussion

Lung cancer is a leading cause of cancer incidence and mortality worldwide [1]. Accumulating evidence indicates that CSCs are responsible for resistance to conventional therapies due to the stemness properties [51]. Several DUBs have been shown to be important in the regulation of lung cancer stemness. For instance, USP21 deubiquitinates and stabilizes Nanog protein stability [52]. USP17 and OTUD3 promote lung cancer stemness through mediating TRAF2/TRAF3 complex and stabilizing GRP78, respectively [53,54]. Herein, we show that USP4 is critically important in promoting lung cancer stemness via stabilizing Twist1 expression. This notion is supported by the evidence that ectopic expression of USP4, but not USP4^C311A^, significantly upregulates Oct4 and Sox2 expression, increases the population of CD133+ cells and promotes mammosphere formation, all of which can be rescued by simultaneous silencing of Twist1. 

EMT transcriptional factors, Snail/Slug, Twist1 and ZEB1, are pivotal in regulation of tumor metastasis and stemness [38]. It has been reported that expression of these factors is tightly controlled by DUBs. Snail protein can be deubiquitinated and stabilized by USP26, USP11, USP10, USP37 or OTUB1 [55,56,57,58,59]. Slug can be stabilized by USP10 and DUB3 [55,60]. ZEB1 is stabilized by USP51 [61]. Twist1 protein, on the other hand, can be stabilized by deubquitinase DUB3 in response to IL-6 [35,36,37,60]. In this study, we uncover that USP4 is a novel deubquitinase of Twist1 and that silencing of Twist dramatically inhibits USP4-induced stemness of lung cancer cells, suggesting that the USP4–Twist1 axis plays a critical role in lung cancer stemness.

The biological function of USP4 in cancer development appears to be complex. It has been reported that USP4 can deubiquitinate and stabilize TRAF2/TRAF6 to inhibit TNFα-induced cancer cell migration [62]. In addition, USP4 expression could be upregulated by CircBMPR2-miR-553 to suppress breast cancer resistance to tamoxifen [63]. These results suggest a role for USP4 in growth suppression. However, USP4 is highly expressed in various cancers [42]. A wealth of evidence strongly suggests that USP4 functions as a tumor-promoting protein, as exemplified by the ability of USP4 to promote tumorigenesis via deubiquitinating and stabilizing ARF-BP1, β-catenin and TGF-βRI [47,64,65,66]. In addition, USP4 can also promote cell cycle procession and tumorigenesis by deubiquitinating SART3 and Ro52 [45,46]. Our results presented in this study provide another series of strong evidence supporting USP4 as an oncoprotein. 

Importantly, our results showed clear clinical relevance, supported by the observations that expression of USP4 and Twist1 is positively correlated in lung cancers and that high expression of USP4/Twist1 is associated with poor overall survival in lung adenocarcinoma patients. Together, our findings demonstrate that USP4 deubiquitinates and stabilizes Twist1 protein to promote lung cancer stemness. Thus, targeting the USP4–Twist1 axis may represent a novel therapeutic approach for lung cancer treatment. 

## 4. Materials and Methods

### 4.1. Cell Culture

Human non-small cell lung cancer H1975 cells were cultured in RPMI-1640 medium (Hyclone, Logan, UT, USA) supplemented with 10% fetal bovine serum (FBS; Hyclone, Logan, UT, USA), 100 units/mL penicillin (GIBCO, Rockville, MD, USA) and 100 μg/mL streptomycin (GIBCO, Rockville, MD, USA). Human non-small cell lung cancer A549 cells and human embryonic kidney HEK293T cells were cultured in DMEM medium supplemented with 10% fetal bovine serum (FBS; Hyclone, Logan, UT, USA), 100 units/mL penicillin (GIBCO, Rockville, MD, USA) and 100 μg/mL streptomycin (GIBCO, Rockville, MD, USA). Cells were maintained in a humidified 37 °C incubator under a 5% CO_2_ atmosphere.

### 4.2. Plasmids and Lentiviral Infection

Twist1 recombinant expressing vector (EX-U1219-Lv105) was purchased from GENECOPIEA. A pLVX-puro vector was used to generate recombinant lentiviruses expressing human USP4. The USP4^C311A^ mutants were generated by KOD-Plus-Mutagenesis kit (SMK-101, Toyobo Osaka). Short hairpin RNA constructs targeting human USP4 or Twist1 were generated using pLKO.1-puro vector as described [67]. All plasmid constructs used in this study were confirmed by DNA sequencing. The amplification primer and specific shRNA sequences used in this study are listed below:

For Flag-USP4, Forward: 5′ GAGGATCTATTTCCGGTGAAGAGGAG
ATCTGCCGCCGCGA 3′; Reverse: 5′ TCTAGAACTAGTCTCGAGGTTA
AACCTTATCGTCGTCAT 3′. For Flag-USP4^C311A^, Forward: 5′ ACCGCCT
TCATGAACTCCGCTTTGC 3′; Reverse: 5′ GTTTCCCAGGTTTCCAAGTC
CACAG 3′
For anti-USP4, ^#^ 1: CCCAACTGTAAGAAGCATCAA; ^#^ 2 GCCCAGAAT
GTGCTAAGGTTT. For anti-Twist1: GCTGAGCAAGATTCAGACC

### 4.3. Plasmid Transfection, Lentiviral Infection and RNA Interference

Cells at 70–80% confluence were transfected with expressing plasmids by Lipofectamine 2000 transfection reagent (Invitrogen, Carlsbad, CA, USA). Recombinant lentiviruses were amplified by co-transfecting psPAX2 and pMD2.G packaging plasmids and lentiviral expressing plasmids using Lipofectamine 2000 in HEK293T. Viruses were collected at 60 h after transfection. Cells at 50% confluence were infected with recombinant lentiviruses in the presence of 10 µg/mL polybrene, followed by 12 h incubation.

### 4.4. Western Blot, Immunoprecipitation and Immunohistochemistry (IHC) Analyses 

For Western blot analysis, cells were collected, washed with cold PBS, and resuspended in EBC_250_ lysis buffer (250 mM NaCl, 50 mM Tris pH 8.0, 0.5% Nonidet P-40, 50 mM NaF, 1 mM phenylmethylsulfonyl fluoride, 2 μg/mL aprotinin, and 2 μg/mL leupeptin). Equal amounts of total protein were loaded, separated by SDS-PAGE, transferred to PVDF membranes (Millipore, Darmstadt, Germany), and hybridized to an appropriate primary antibody and HRP-conjugated secondary antibody for subsequent detection by enhanced chemiluminescence. The immunoblots were quantitated by Image Lab 5.0 and normalized to the loading control GAPDH.

Immunoprecipitation and immunohistochemistry analyses were performed according to procedures described previously [68,69]. Human tumor tissue microarray slides (Cat# HLugA030PG02) were purchased from Outdo Biotech Co., (Shanghai, China). Slides were scanned by NanoZoomer (Hamamatsu, Japan) and images were quantified by integrated optical density (IOD) using Image-Pro Plus 6.0 software from Media Cybernetics Inc. (Rockville, MD, USA). Average optical density (AOD) was calculated using the formula: AOD = IOD/Area. Antibodies for USP4 (612819, 1:500 for WB, 1:100 for IHC) and Oct4 (618077, 1:1000 for WB) were purchased from zenbioscience (Chengdu, China). Antibody for Sox2 (AB5603, 1:500 for WB) was purchased from Merck (Darmstadt, Germany). Antibodies for GAPDH (AB0037, 1:1000 for WB) and Twist1 (CY2578, 1:1000 for WB, 1:200 for IHC) were purchased from Abways (Shanghai, China). Antibody for Flag (14793, 1:1000 for WB) was purchased from Cell Signaling Technology (Danvers, MA, USA).

### 4.5. Tumorspheres Formation Assay

20,000 cells were seeded on the 6-ultral-low-adherient plates and grown in DMEM/F12 medium containing 5 mg/mL insulin (Sigma, Taufkirchen, Germany), 2% B27 (Invitrogen, Carlsbad, CA, USA), 20 ng/mL epidermal growth factor (R&D systems), 100 units/mL penicillin (GIBCO, Rockville, MD, USA) and 100 μg/mL streptomycin (GIBCO, Rockville, MD, USA) for 7 days. Tumorspheres were defined to the diameter of spheres that were greater than 50 μm. The number of tumorspheres of each well were counted, normalized and presented to reflect tumorsphere formation ability.

### 4.6. Quantitative PCR 

Total RNA was extracted from cells using RNeasy plus Mini Kit (cat# 74134, QIAGEN, Germantown, MD, USA) and reverse-transcribed according to the manufacturer’s instructions. Q-PCR was performed for Twist1 and GAPDH. The Q-PCR reactions were carried out in CFX-960 Real time PCR System (Bio-Rad, CA, USA) and using Bio-Rad SoFast Eva-Green Supermix (Bio-Rad, Hercules, CA, USA) according to the manufacturer’s instructions. Relative quantitation values were calculated using the ΔΔCt method. Q-PCR primer for Twist1, Forward: GTCCGCAGTCTTACGAGGAG; Reverse: GCTTGAGGGTC

TGAATCTTGCT. For GAPDH, Forward: TGGACTCCACGACGTACTCA; Reverse: AATCCCATCACCATCTTCCA.

### 4.7. Flow Cytometry Assay 

To analyze CD133+ cell population, cells were harvested and washed twice with PBS, then were re-suspended in staining buffer (Bio-Rad), and cell numbers were counted. Cells were incubated with Phycoerythrin (PE) conjugated anti-CD133 antibody (12-1338-42, Invitrogen, Carlsbad, CA, USA) or PE conjugated IgG1 kappa isotype control antibody (12-4714-81, Invitrogen, Carlsbad, CA, USA) for 30 min at 4 °C. Cells were then washed twice with staining buffer and re-suspended by staining buffer, and analyzed by FACS machine (Calibur, Bio-Rad, Hercules, CA, USA). The data of flow cytometry assay were analyzed by FlowjoX software.

### 4.8. Clinical Relevance Analysis

Pearson correlation of USPs and Oct4/Sox2 expression was analyzed using Oncomine dataset “Bhattacharjee Lung”. The mRNA levels of USP4 in normal human lung tissues and lung cancers were analyzed using Oncomine dataset “Gaber lung”. The mRNA levels of USP4 in stage I or stage II–IV human lung cancers were analyzed using Oncomine dataset “Bild lung”. The mRNA levels of USP4 in 3 year-alive or 3 year-dead human lung cancer patients were analyzed using Oncomine dataset “Raponi lung”. Kaplan–Meier plots of overall survival of human lung cancer patients stratified by the USP4 or Twist1 mRNA expression levels were analyzed using the Kaplan–Meier survival datasets. The high and low groups were defined by the best cutoff.

Gene signature enrichment analyses (GSEA) were performed by GSEA software (v 4.0.3, UC San Diego and Broad Institute Inc., San Diego, CA, USA) based on GSE19804 dataset available on GEO database. The mean value of each gene expression involved in the assay was used to divided into “high” and “low” groups. Notch and TNF signaling related genes were described in Figure S6.

### 4.9. Statistical Analysis

GraphPad Prism 6.0 (GraphPad Software Incorporation, San Diego, CA, USA) was used for data recording, collection, processing, and calculation. All cell-based experiments were performed at least three times in duplicates. Data were presented as means ± SD. Quantitative data were analyzed statistically using Student’s *t*-test to assess significance.

## 5. Conclusions

Our results demonstrate that deubiquitinase USP4 promotes lung cancer cell stemness via upregulation of Twist1, Oct4 and Sox2 expression. Mechanistically, we have identified USP4 as a novel deubiquitinase of Twist1. In addition, USP4 expression is elevated in human lung cancer specimens and is positively correlated with Twist1 expression. High expression of USP4/Twist1 is associated with poor clinical outcomes of lung cancer patients. Thus, targeting USP4 may represent a novel therapeutic approach for lung cancer treatment.

## Supplementary Materials

The following are available online at https://www.mdpi.com/2072-6694/12/6/1582/s1, Figure S1: Correlation of USPs and Oct4/Sox2 expression in lung adenocarcinoma; Figure S2: USP4 significantly regulates proteins expression of Oct4 and Sox2; Figure S3: Silencing of USP4 downregulates Twist1, Oct4 and Sox2 expression and inhibits stemness of lung cancer A549 cells; Figure S4: Ectopic expression of USP4 upregulates Twist1, Oct4 and Sox2 expression and promotes stemness of lung cancer A549 cells; Figure S5: Ectopic expression of USP4 increases Oct4 and Sox2 protein levels via upregulation of Twist1 protein expression; Figure S6: Genes related to TNF and Notch signaling used for Gene signature enrichment analysis.
