# Supplementary material for: The Deubiquitinase USP4 Stabilizes Twist1 Protein to Promote Lung Cancer Cell Stemness
Source: Cancers (Basel). 2020 Jun 15;12(6):1582. doi: 10.3390/cancers12061582 (PMC7352958; doi:10.3390/cancers12061582)
Supplement: Supplementary file 1 [file cancers-12-01582-s001.zip › Suppmentary materials/cancers-802641-suppl proof-V2.docx]

Supplementary Materials: The Deubiquitinase USP4 Stabilizes Twist1 Protein to Promote Lung Cancer Cell Stemness


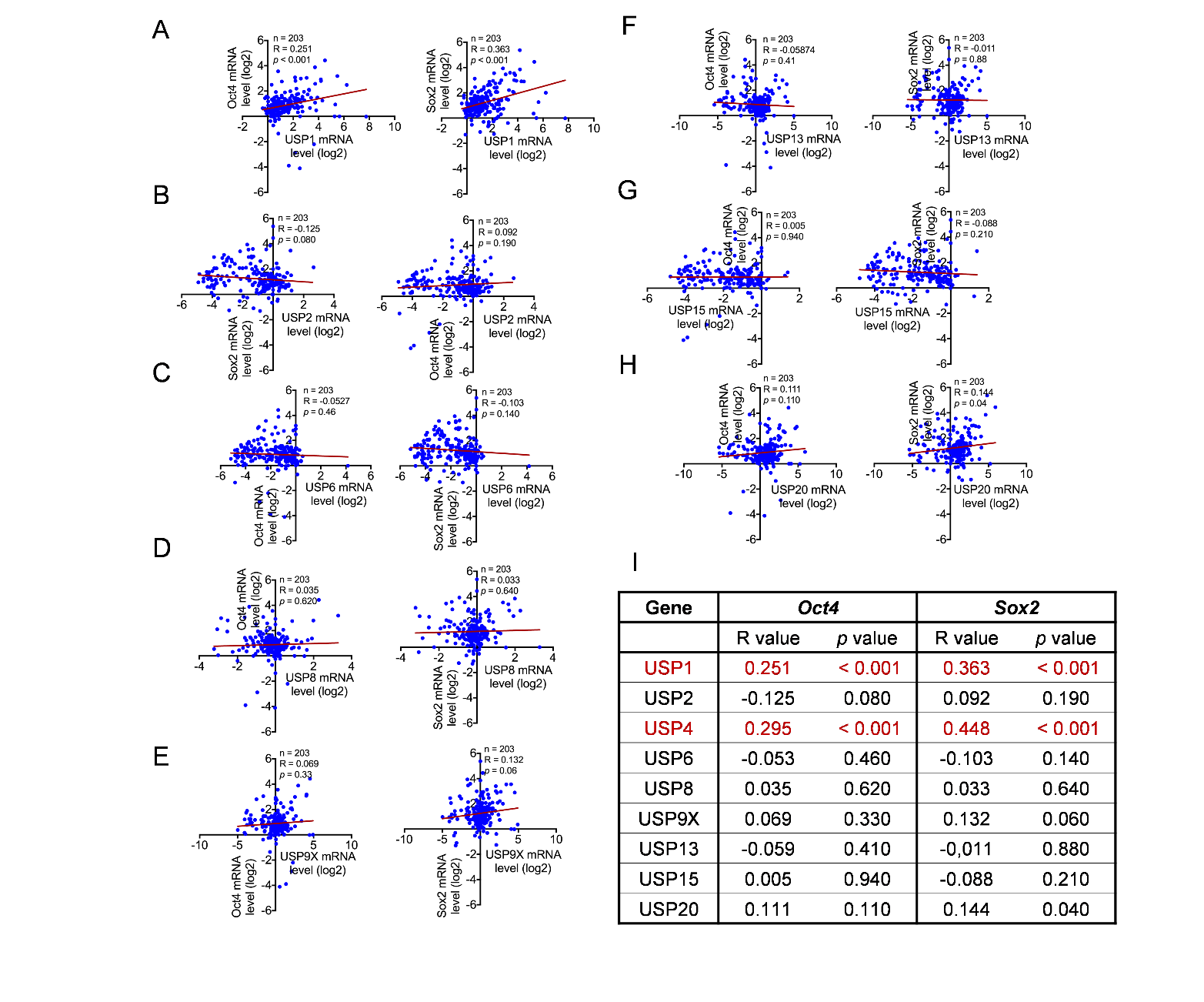


**Figure S1.** Correlation of USPs and Oct4/Sox2 expression in lung adenocarcinoma. (**A**–**H**) The Oncomine dataset "Bhattacharjee Lung" was used to analyze Pearson correlation of USPs and Oct4/Sox2 expression. (**I**) Table summarized the data derived from Person correlation analyses.


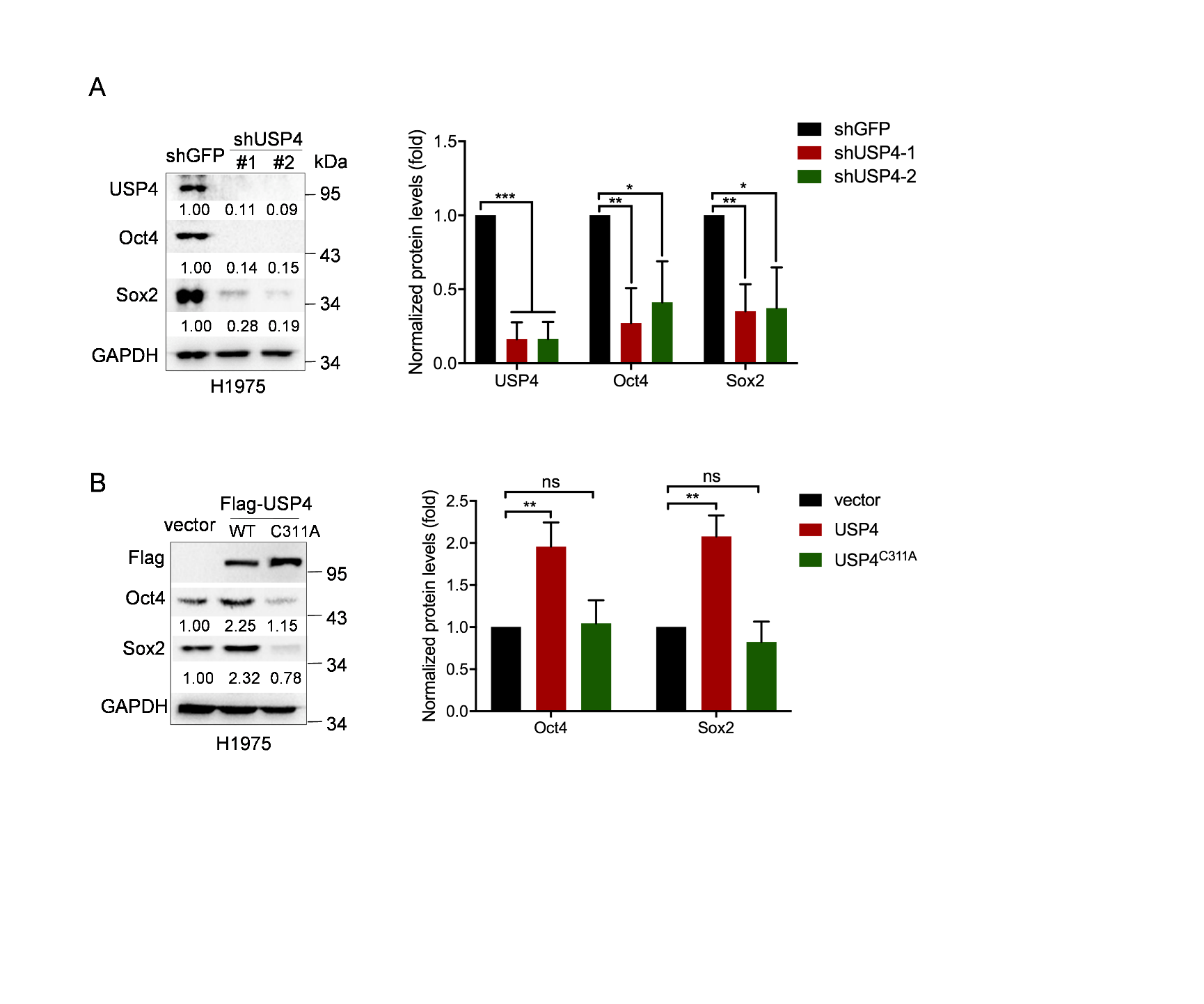


**Figure S2.** USP4 significantly regulates proteins expression of Oct4 and Sox2. (**A**) H1975 cells stably expressing two different shRNAs against USP4 (shUSP4-#1 or shUSP4-#2) were subjected to Western blot analyses. The immunoblots were quantitated and normalized to the loading control GAPDH. Data from three independent experiments were presented as means ± SD. * *p* < 0.05, ** *p* < 0.01, *** *p* < 0.001. (**B**) H1975 cells stably expressing Flag-USP4 or Flag-USP4^C311A^ were subjected to Western blot analyses. The immunoblots were quantitated and normalized to the loading control GAPDH. Data from three independent experiments were presented as means ± SD. ** *p* < 0.01.


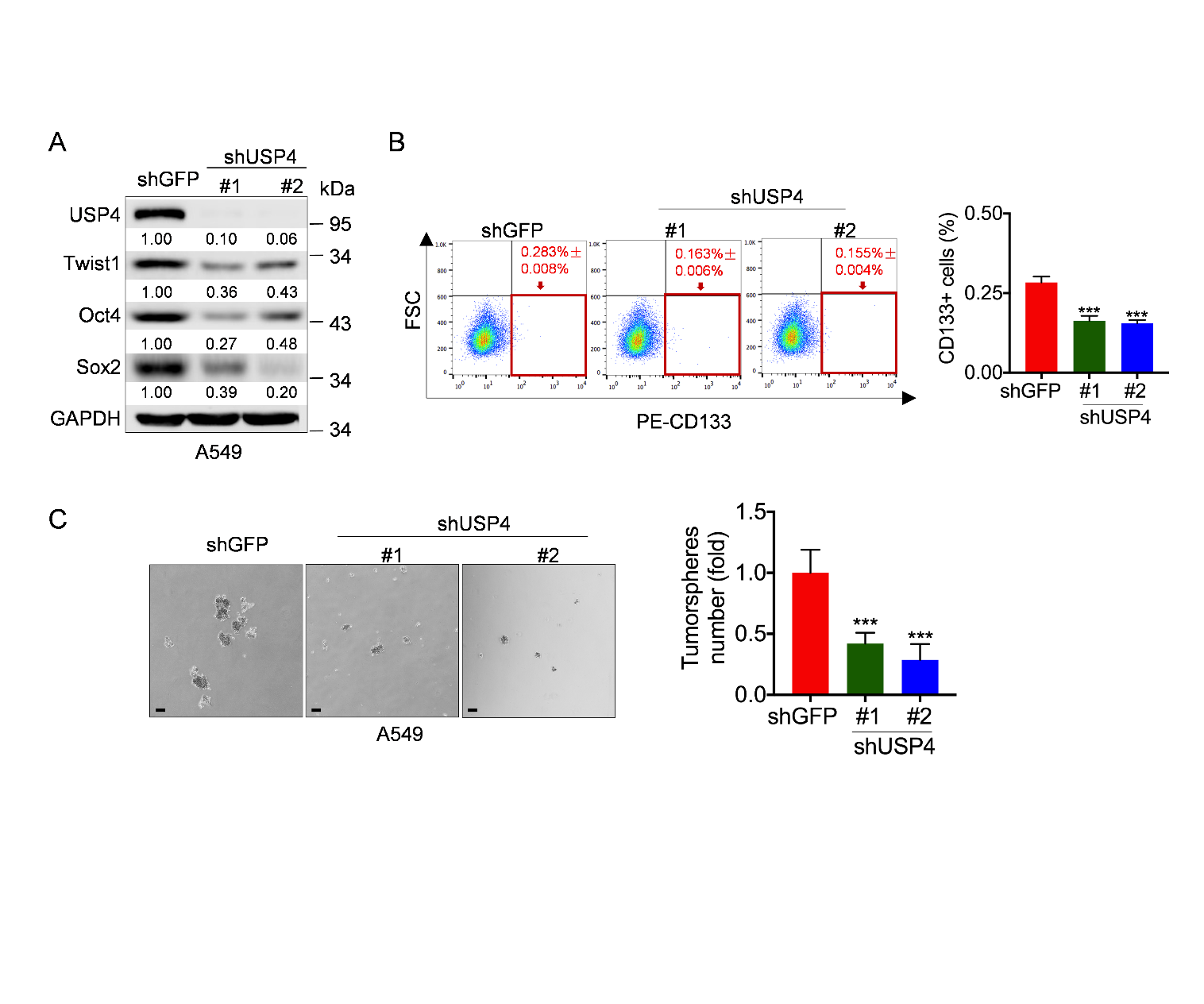


**Figure S3.** Silencing of USP4 downregulates Twist1, Oct4 and Sox2 expression and inhibits stemness of lung cancer A549 cells. (**A-C**) A549 cells stably expressing shRNA against USP4 (shUSP4-#1 or shUSP4-#2) were subjected to (**A**) Western blot analyses, (**B**) FACS analyses for CD133-stained cells or (**C**) tumorsphere formation assay. Respective images and quantitation were shown. Data from three independent experiments in duplicates were presented as means ± SD. *** *p* < 0.001. Scale bar = 100 μm.

**
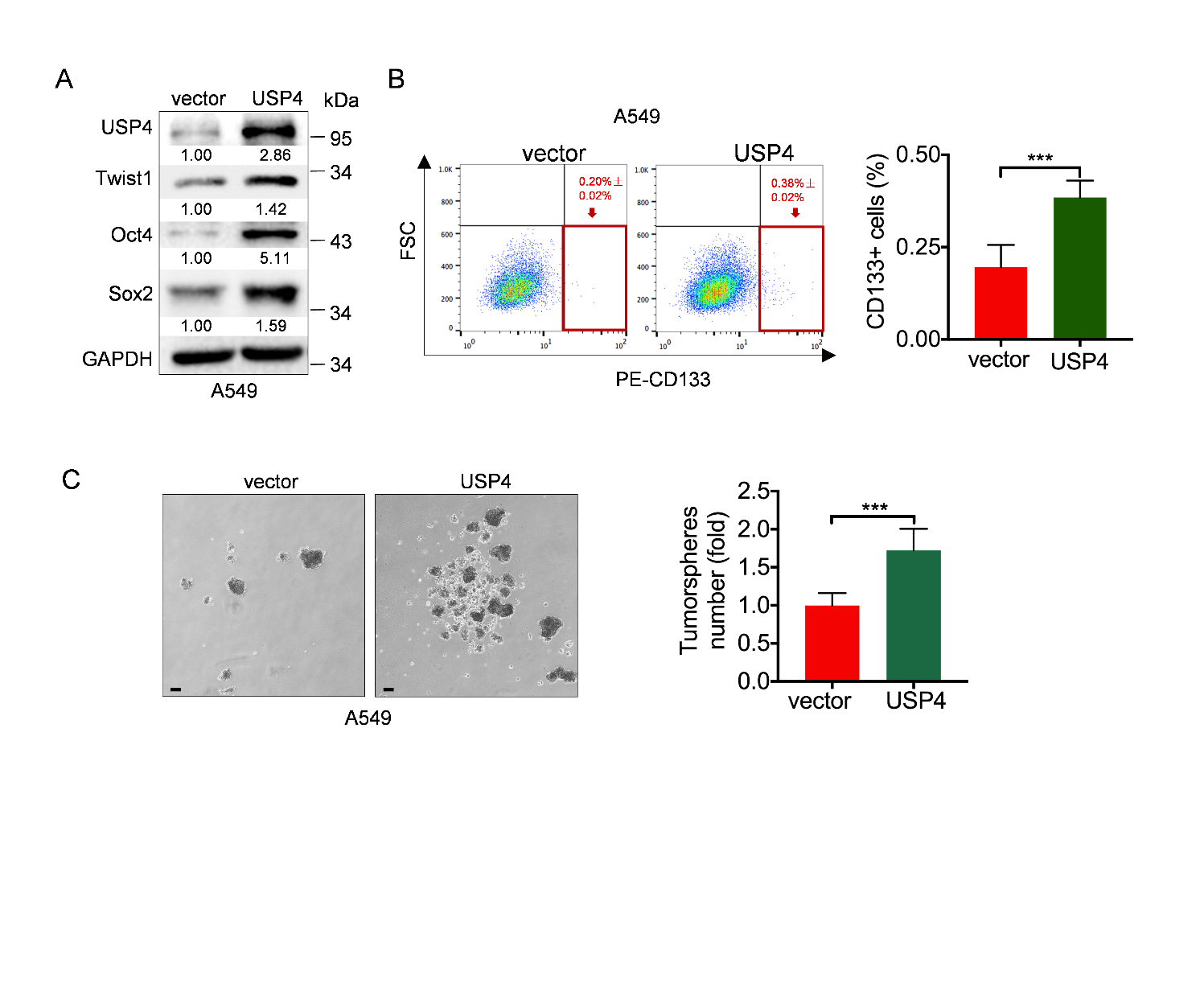
 Figure S4.** Ectopic expression of USP4 upregulates Twist1, Oct4 and Sox2 expression and promotes stemness of lung cancer A549 cells. A549 cells stably expressing Flag-USP4 or a vector control were subjected to (**A**) Western blot analyses, (**B**) FACS analyses for CD133-stained cells or (**C**) tumorsphere formation assay. Respective images and quantitation were shown. Data of FACS analyses from three independent experiments in triplicates were presented as means ± SD. Data of tumorsphere formation assays from three independent experiments in duplicates were presented as means ± SD. *** *p* < 0.001. Scale bar = 100 μm.


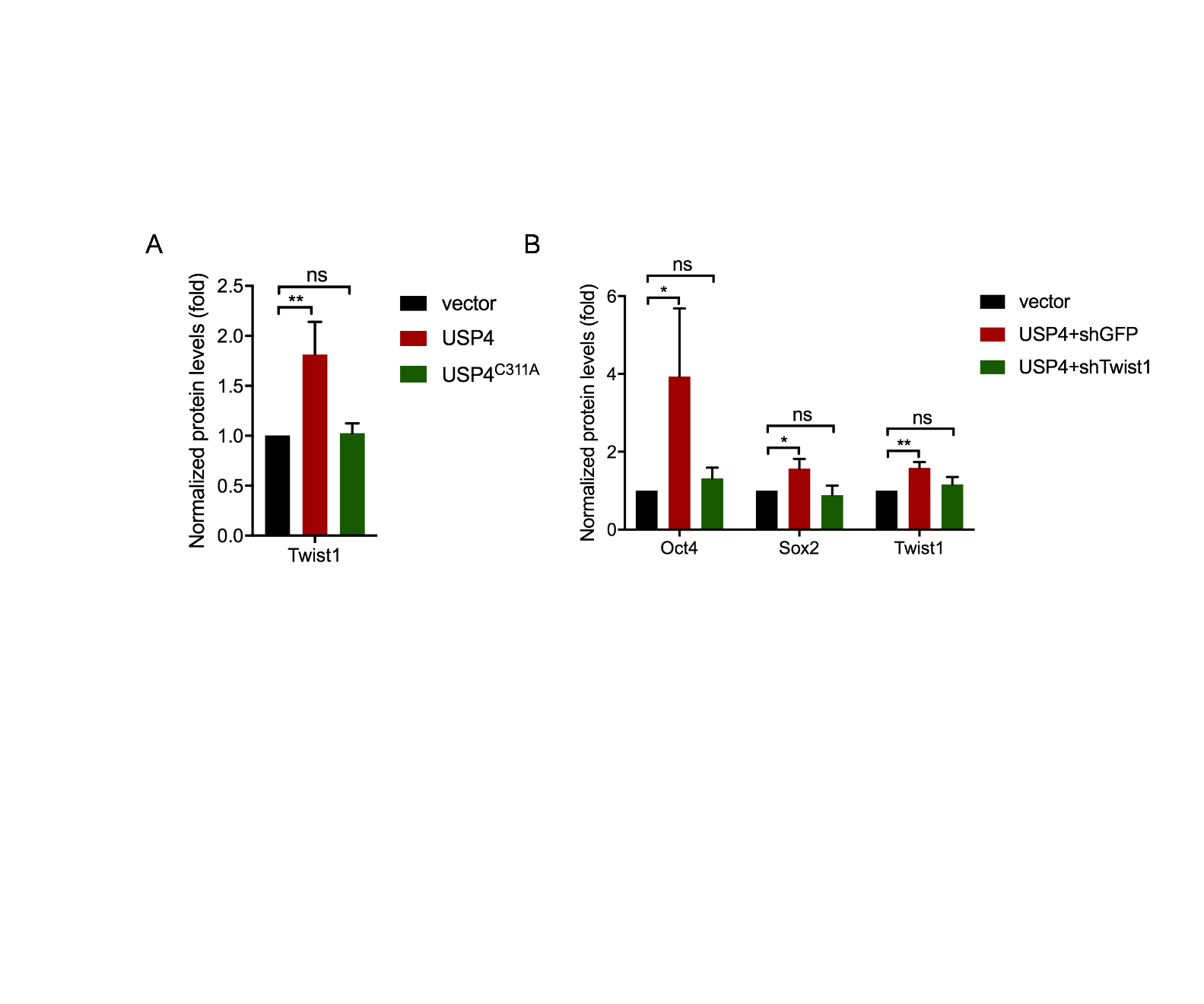


**Figure S5.** Ectopic expression of USP4 increases Oct4 and Sox2 protein levels via upregulation of Twist1 protein expression. (**A**) H1975 cells stably expressing Flag-USP4 or Flag-USP4^C311A^ were subjected to Western blot analyses. The immunoblots were quantitated and normalized to the loading control GAPDH. Data from three independent experiments were presented as means ± SD. ** p< 0.01. (**B**) H1975 cells stably expressing Flag-USP4 and either shTwist1 or shGFP were subjected to Western blot analyses. The immunoblots were quantitated and normalized to the loading control GAPDH. Data from three independent experiments were presented as means ± SD. * *p* < 0.05, ** *p* < 0.01.


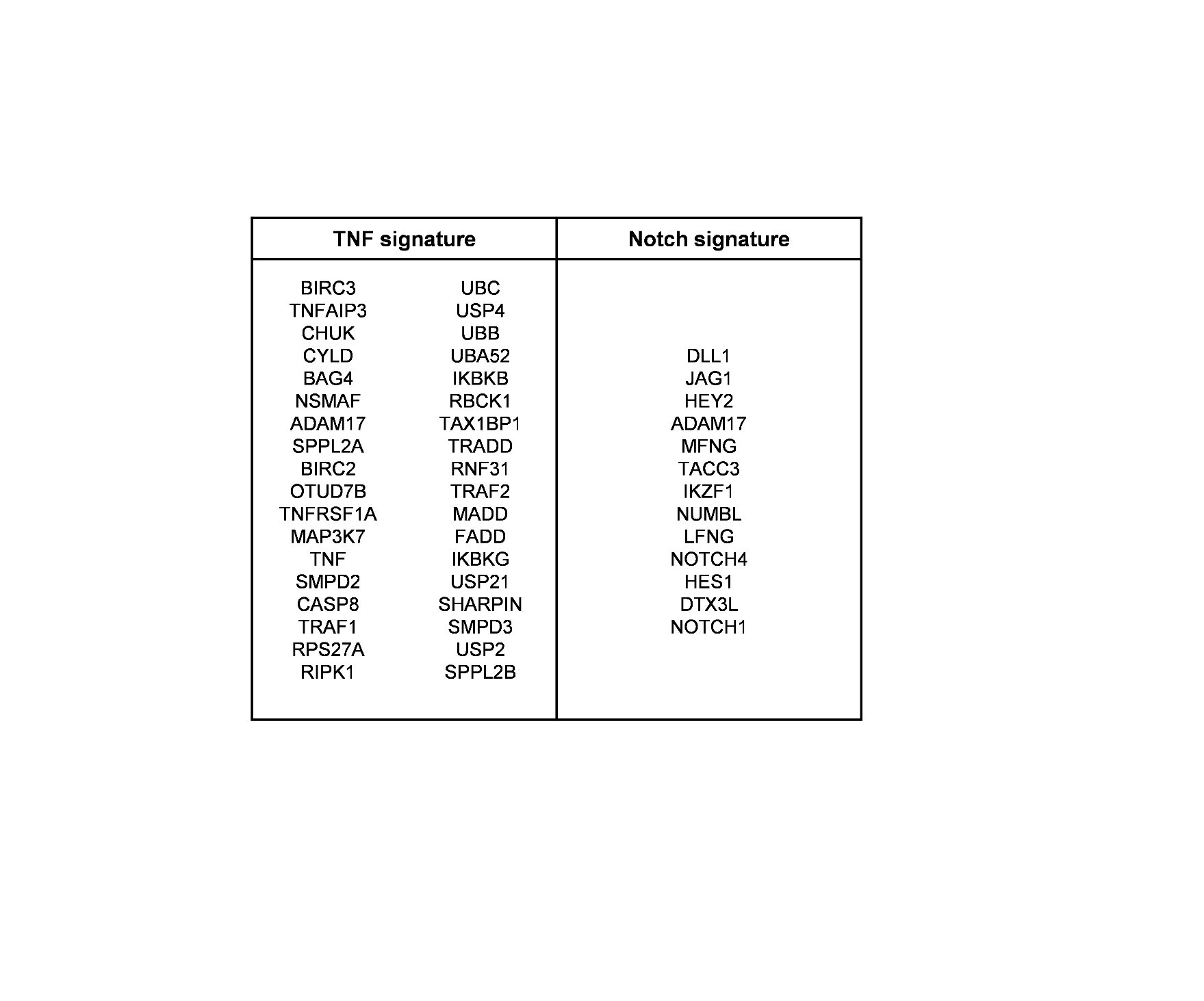


**Figure S6.** Genes related to TNF and Notch signaling used for Gene signature enrichment analysis. 36 genes related to TNF signaling (PMID: 22435550) and 13 key genes in the Notch signaling pathway were used for GSEA. Details described in Materials and Methods.

| 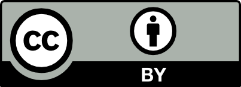 | © 2020 by the authors. Submitted for possible open access publication under the terms and conditions of the Creative Commons Attribution (CC BY) license (http://creativecommons.org/licenses/by/4.0/). |
| --- | --- |
