# Supplementary figures and images for: The Deubiquitinase USP4 Stabilizes Twist1 Protein to Promote Lung Cancer Cell Stemness
Source: Cancers (Basel). 2020 Jun 15;12(6):1582. doi: 10.3390/cancers12061582 (PMC7352958; doi:10.3390/cancers12061582)

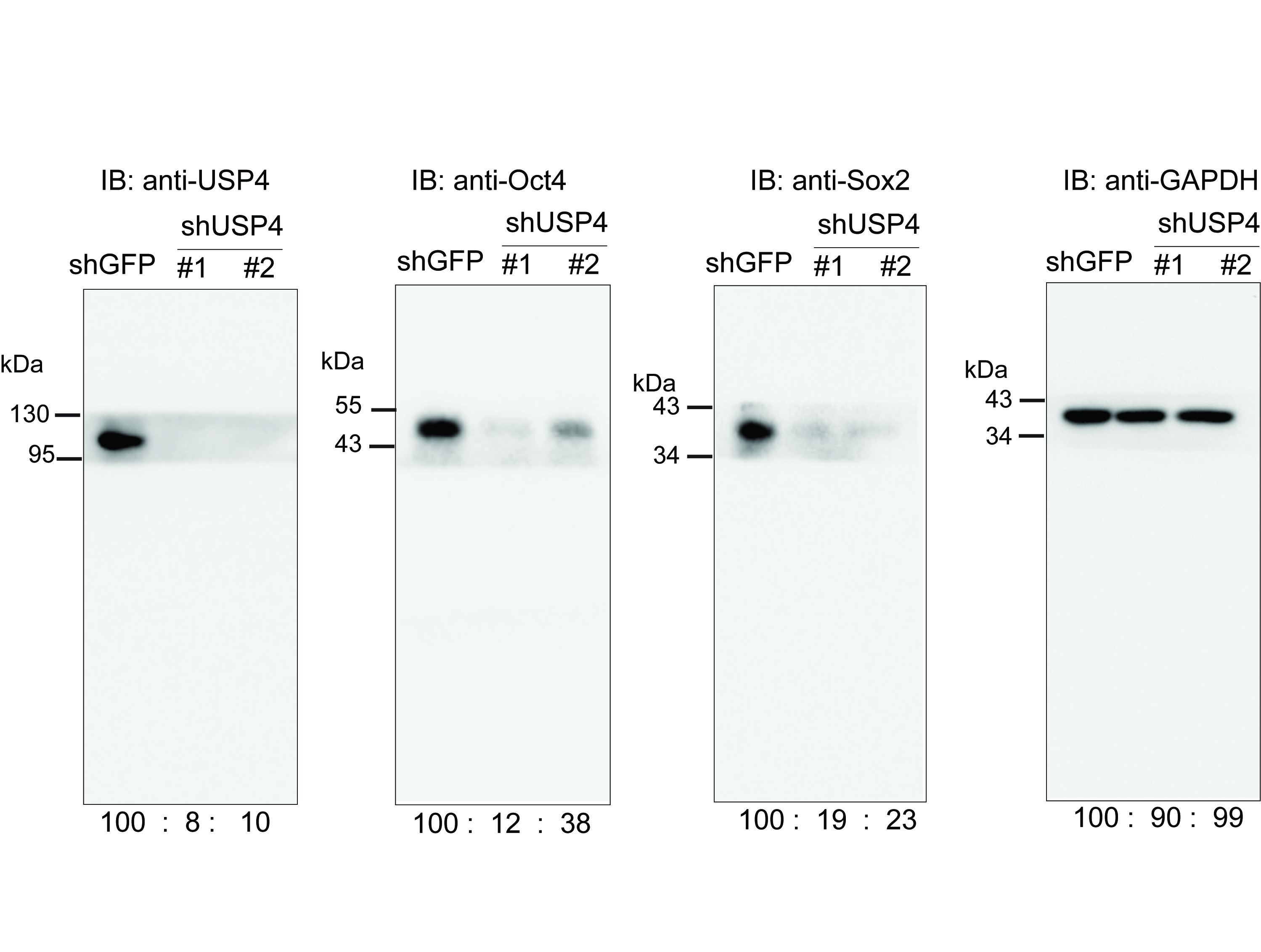

Supplement: Supplementary file 1 [file cancers-12-01582-s001.zip › Suppmentary materials/revised-original-WB-figures/Figure-1B.tif]

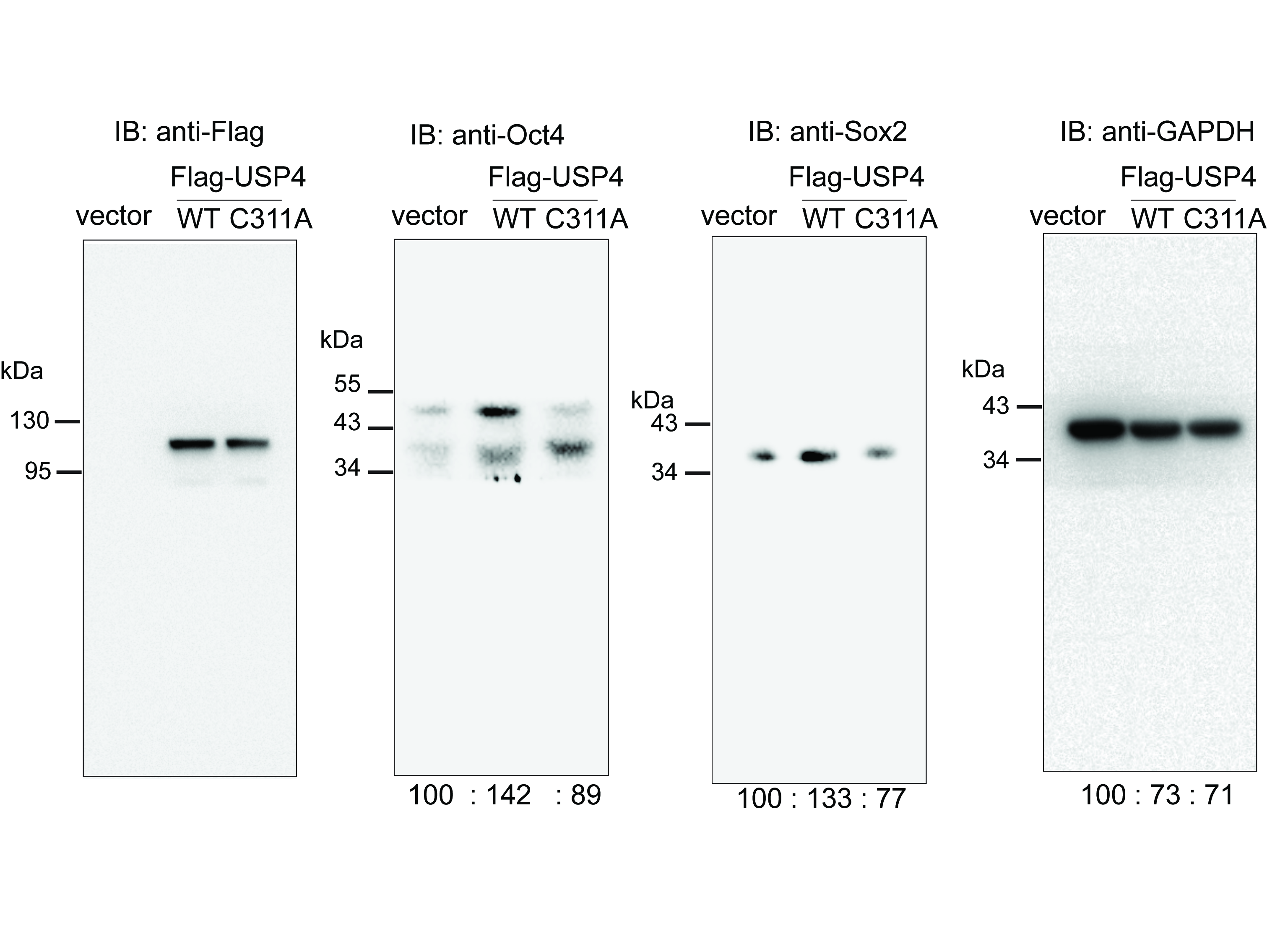

Supplement: Supplementary file 1 [file cancers-12-01582-s001.zip › Suppmentary materials/revised-original-WB-figures/Figure-1F.tif]

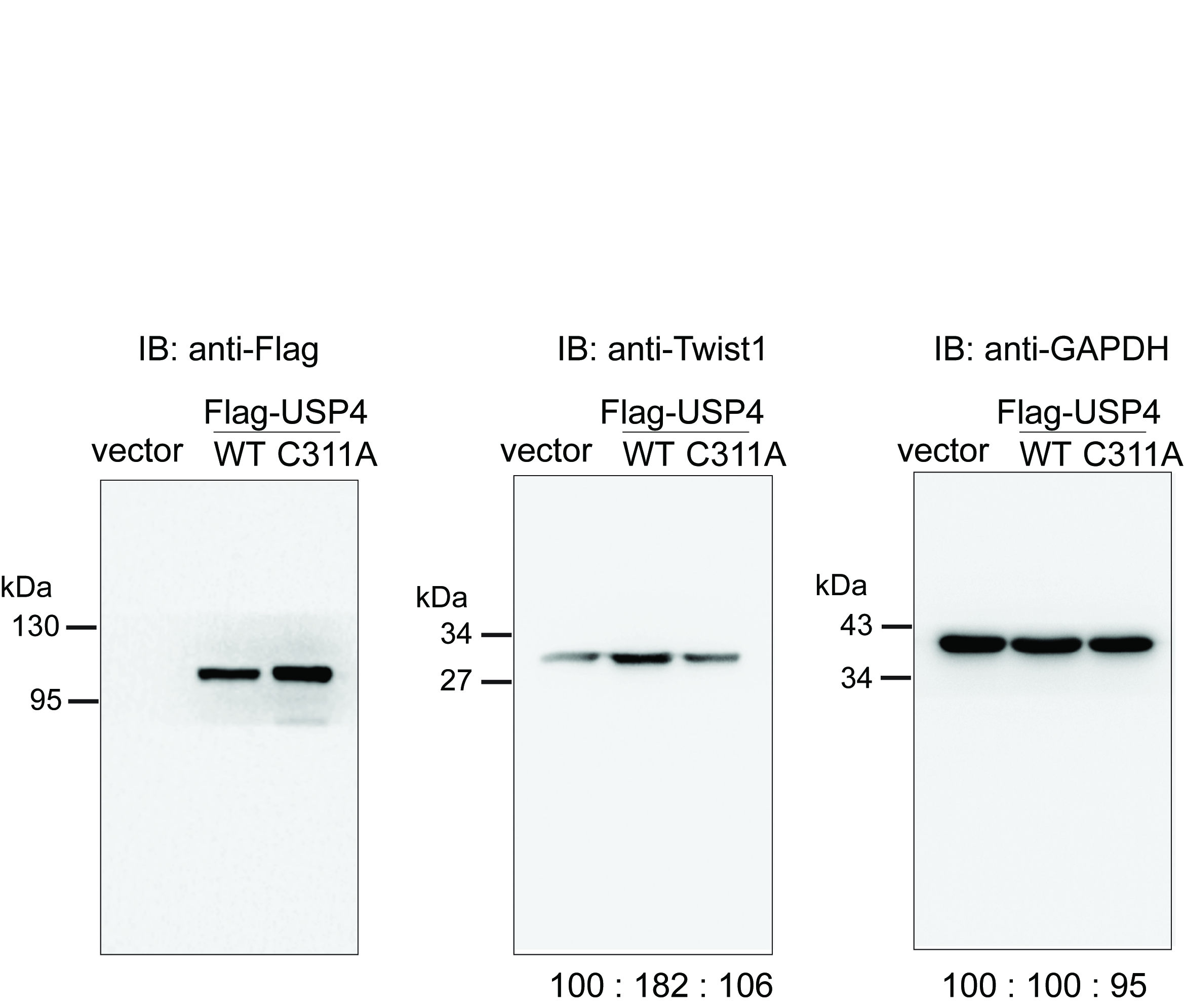

Supplement: Supplementary file 1 [file cancers-12-01582-s001.zip › Suppmentary materials/revised-original-WB-figures/Figure-2B.tif]

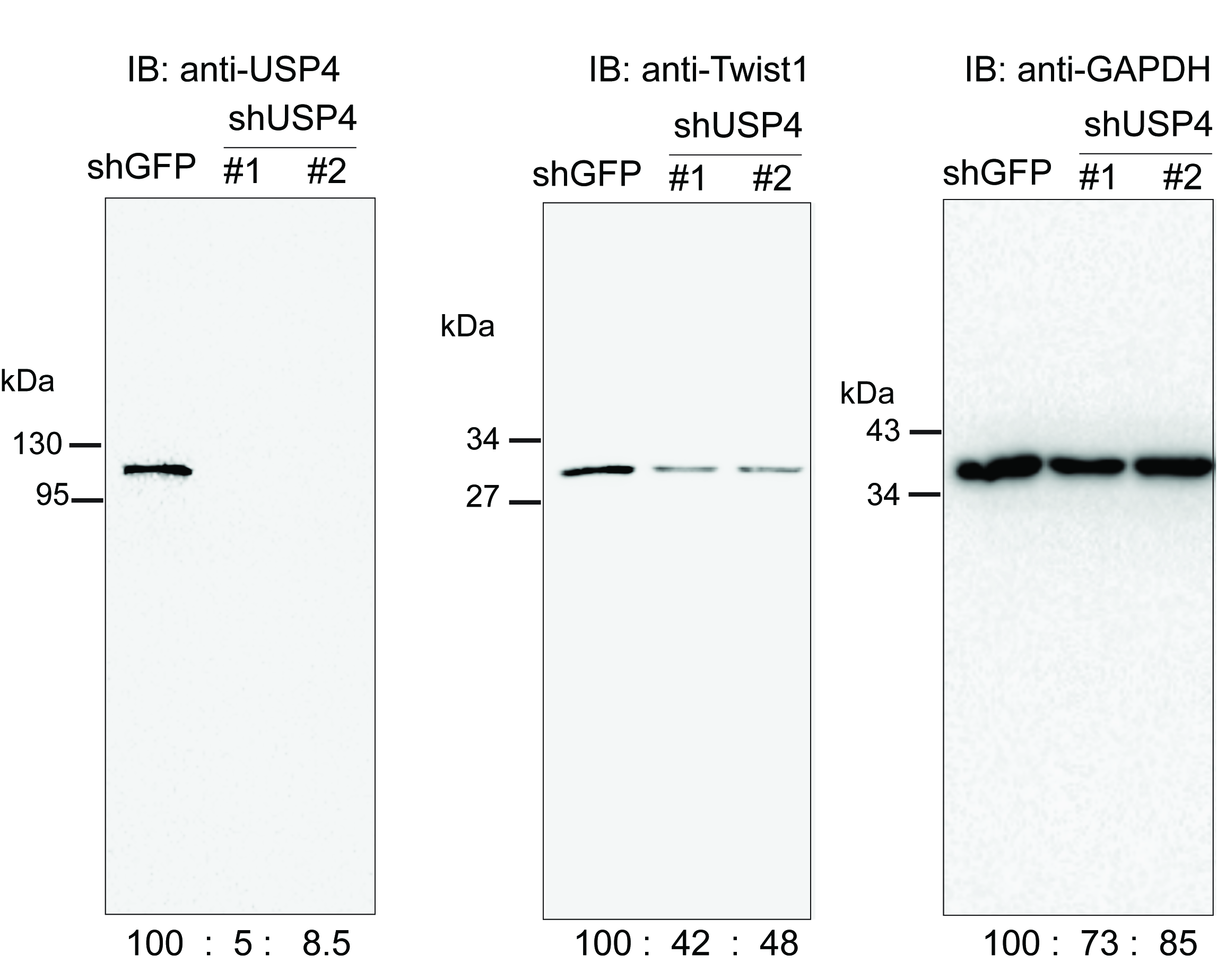

Supplement: Supplementary file 1 [file cancers-12-01582-s001.zip › Suppmentary materials/revised-original-WB-figures/Figure-2C.tif]

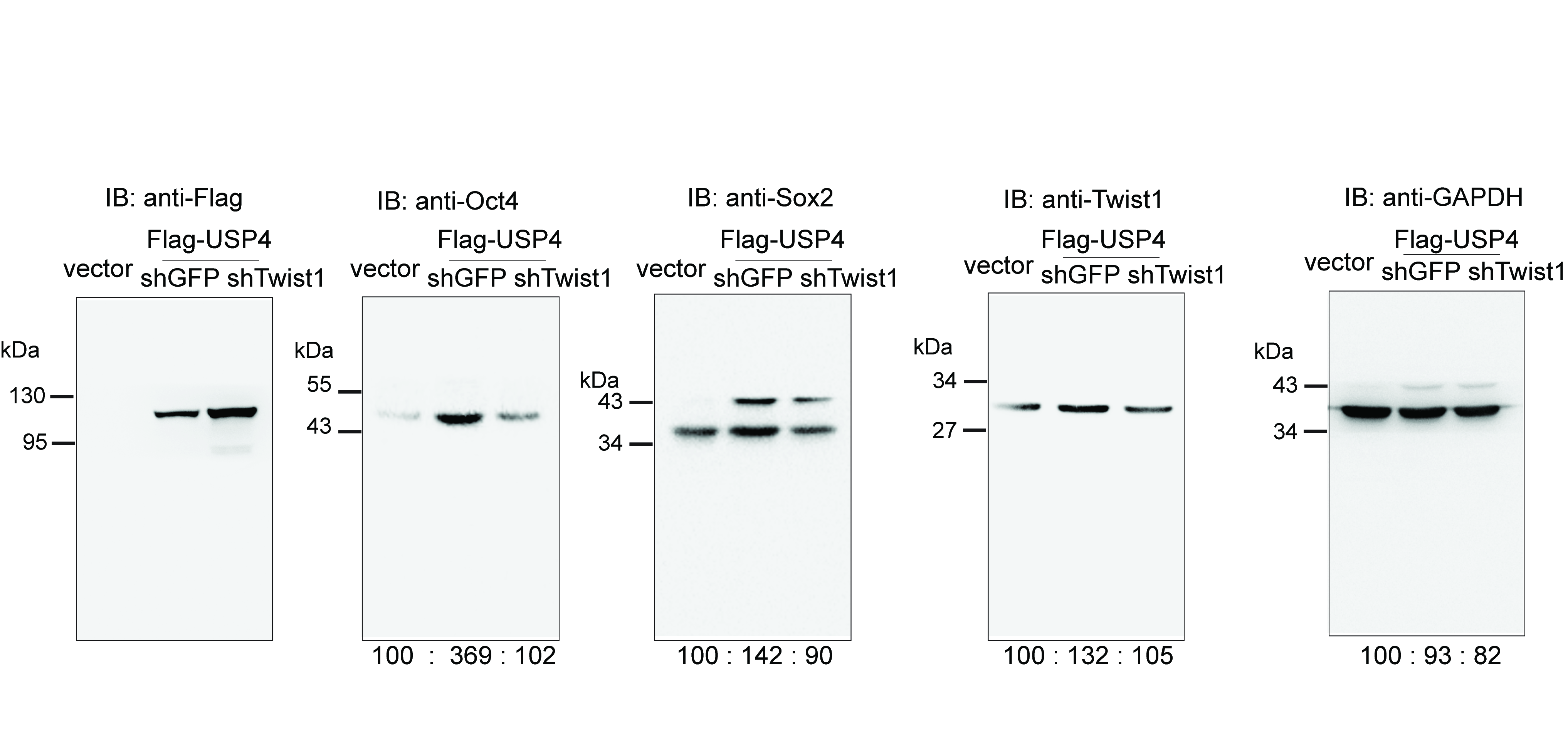

Supplement: Supplementary file 1 [file cancers-12-01582-s001.zip › Suppmentary materials/revised-original-WB-figures/Figure-2D.tif]

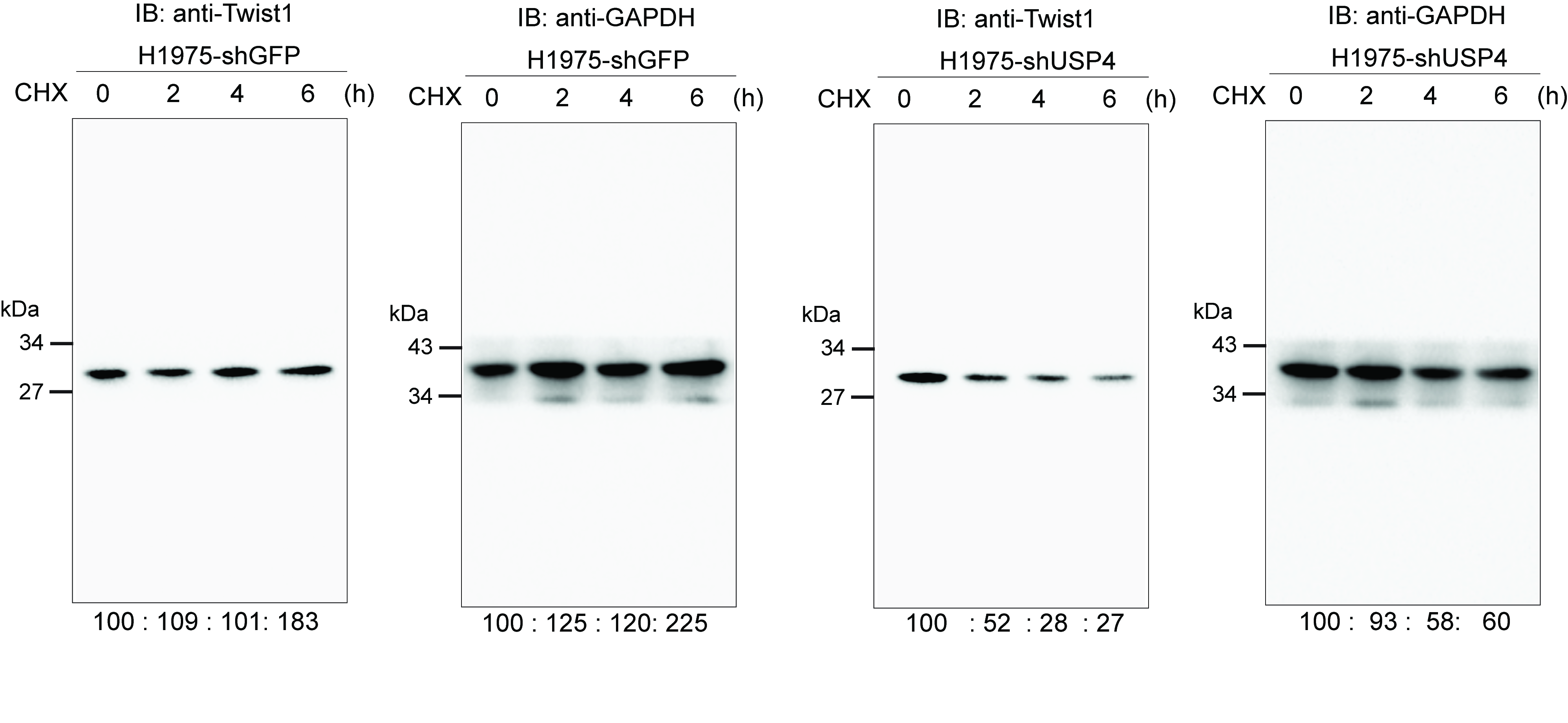

Supplement: Supplementary file 1 [file cancers-12-01582-s001.zip › Suppmentary materials/revised-original-WB-figures/Figure-3B.tif]

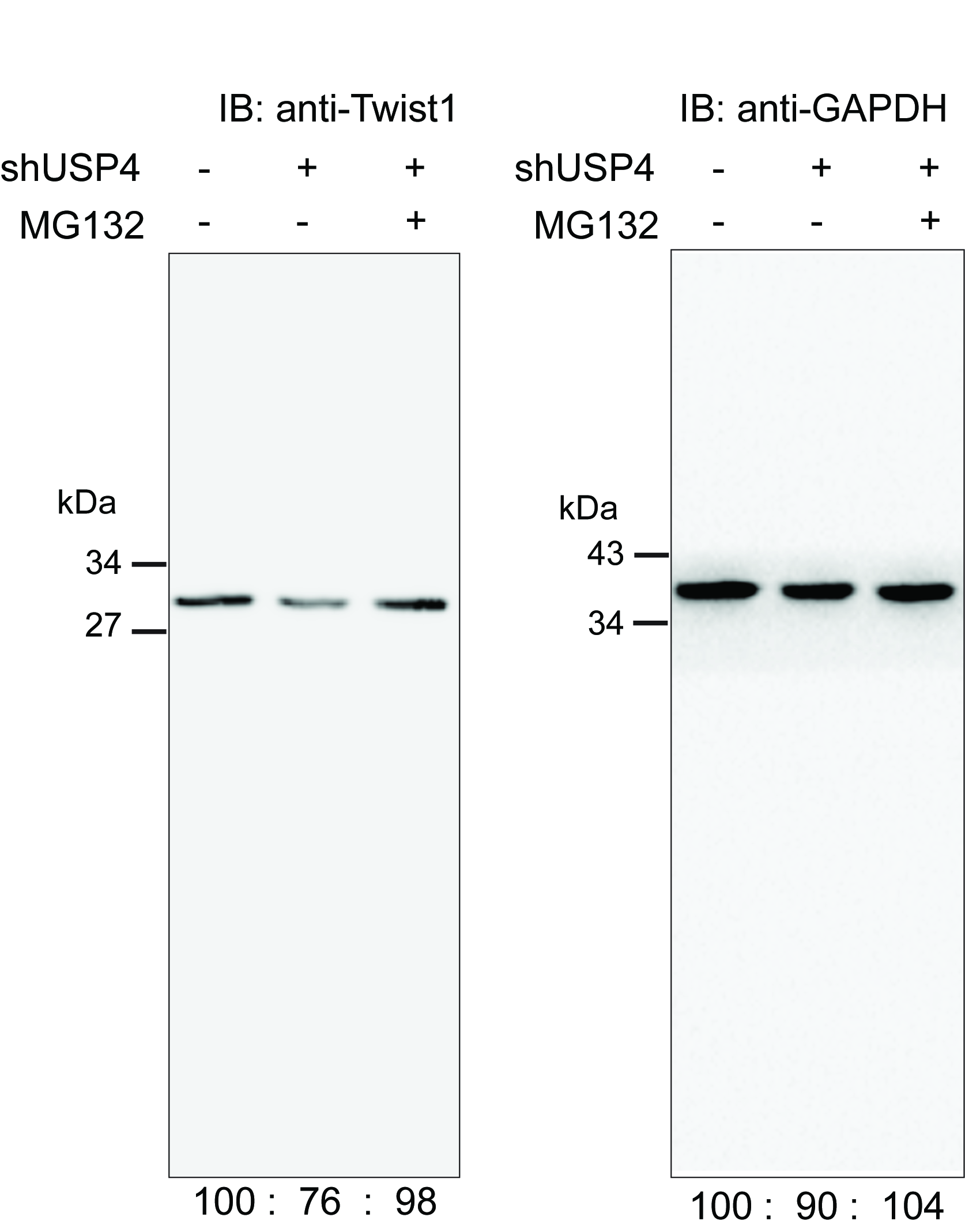

Supplement: Supplementary file 1 [file cancers-12-01582-s001.zip › Suppmentary materials/revised-original-WB-figures/Figure-3C.tif]

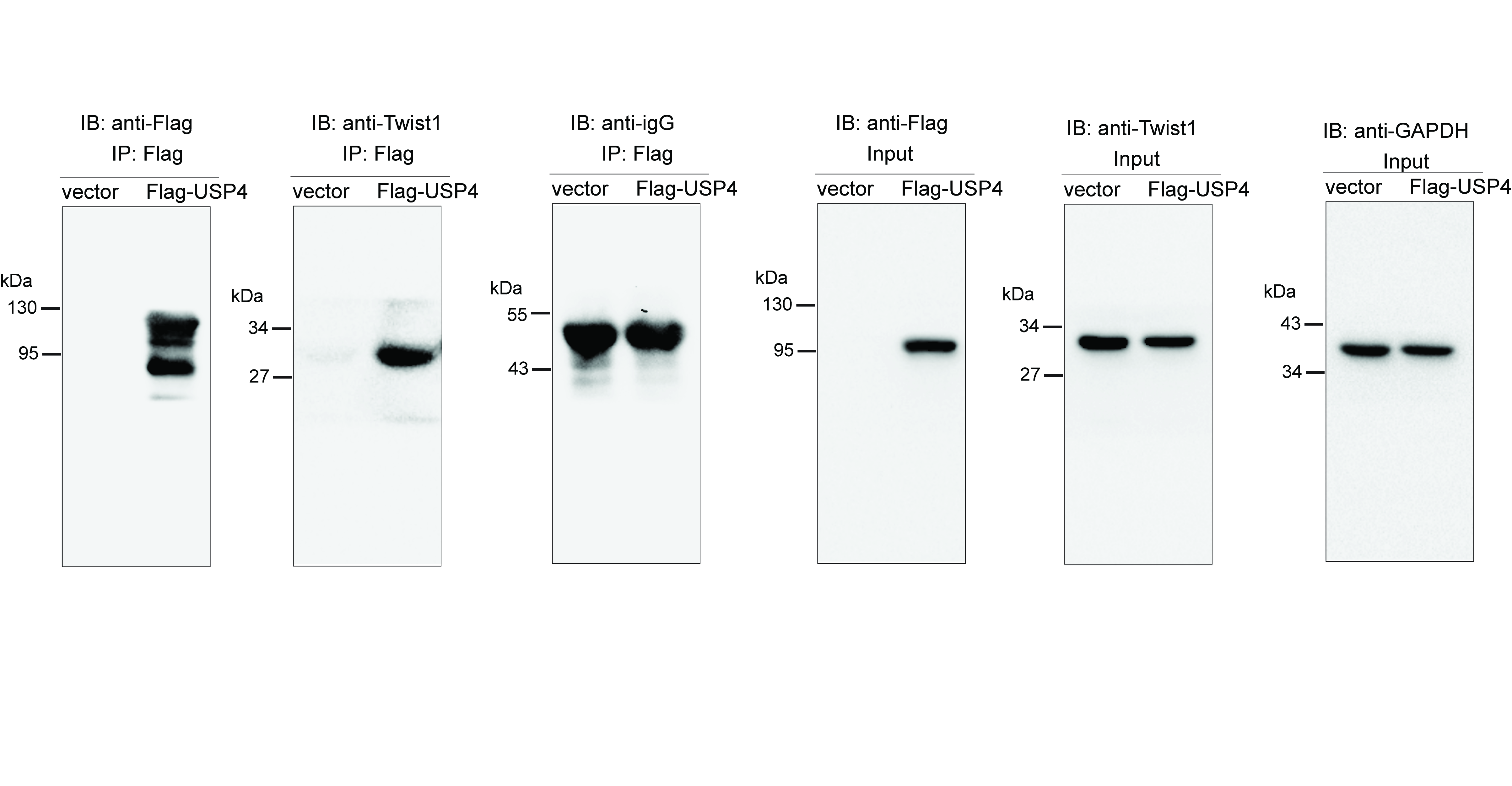

Supplement: Supplementary file 1 [file cancers-12-01582-s001.zip › Suppmentary materials/revised-original-WB-figures/Figure-3D.tif]

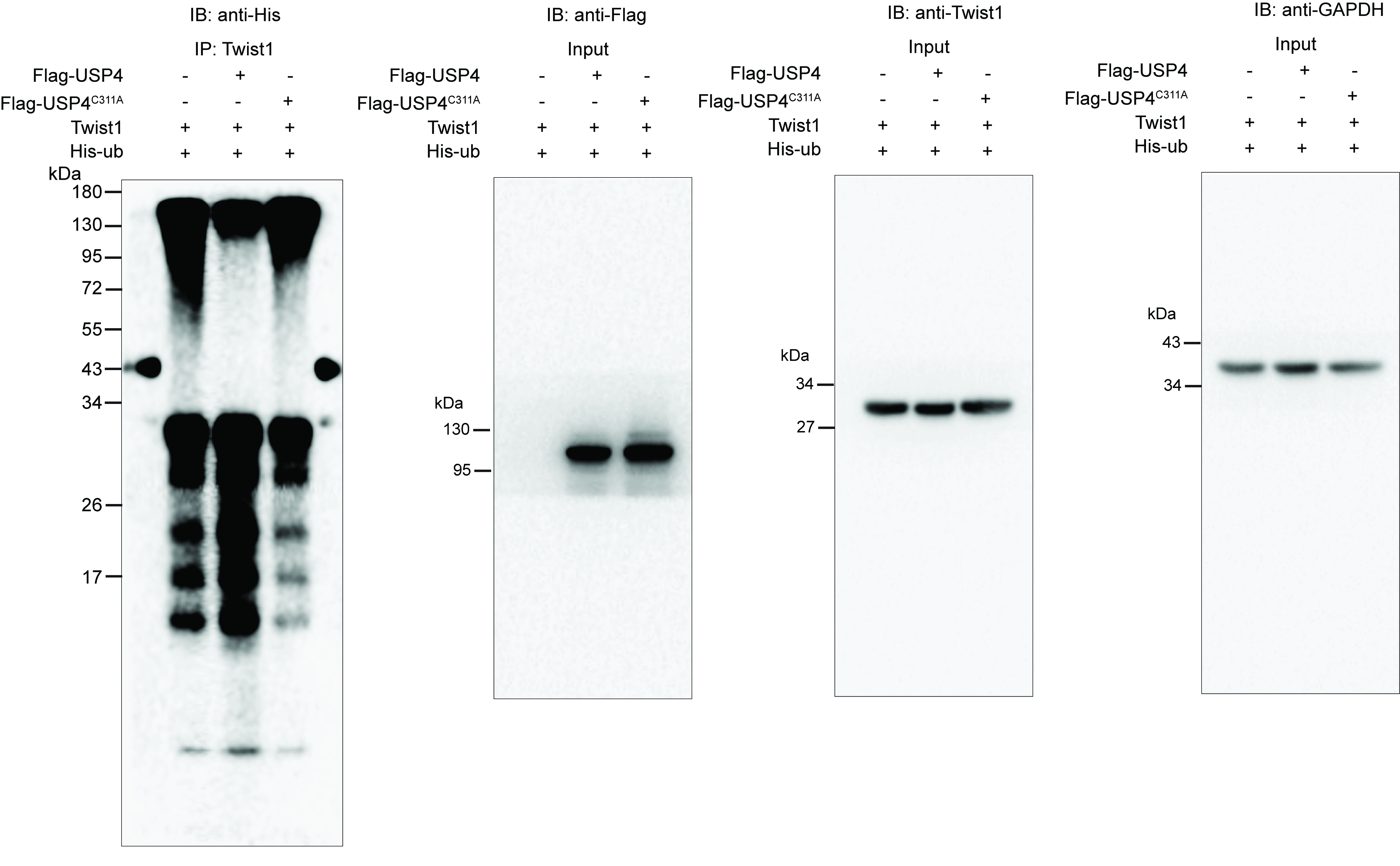

Supplement: Supplementary file 1 [file cancers-12-01582-s001.zip › Suppmentary materials/revised-original-WB-figures/Figure-3E-left.tif]

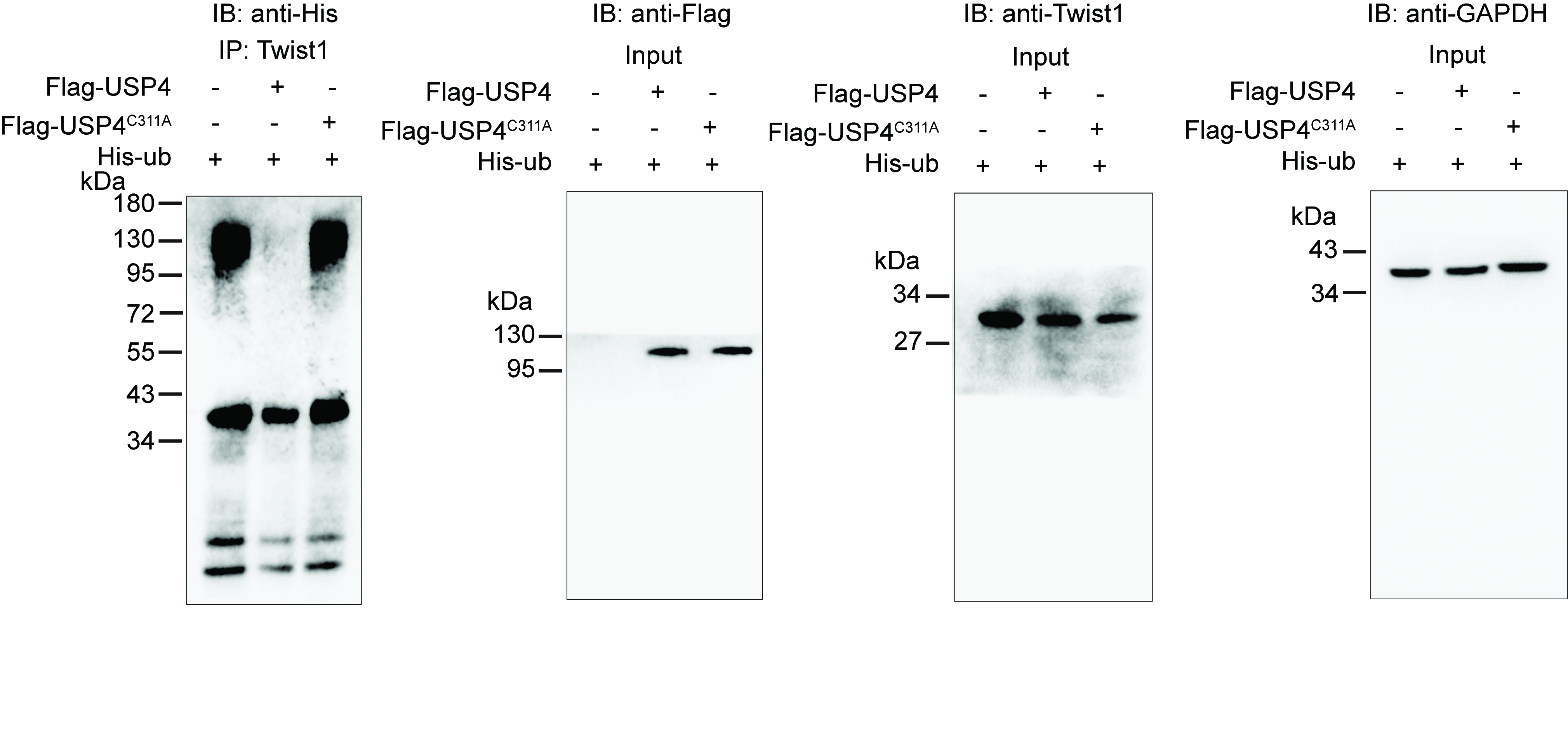

Supplement: Supplementary file 1 [file cancers-12-01582-s001.zip › Suppmentary materials/revised-original-WB-figures/Figure-3E-right.tif]

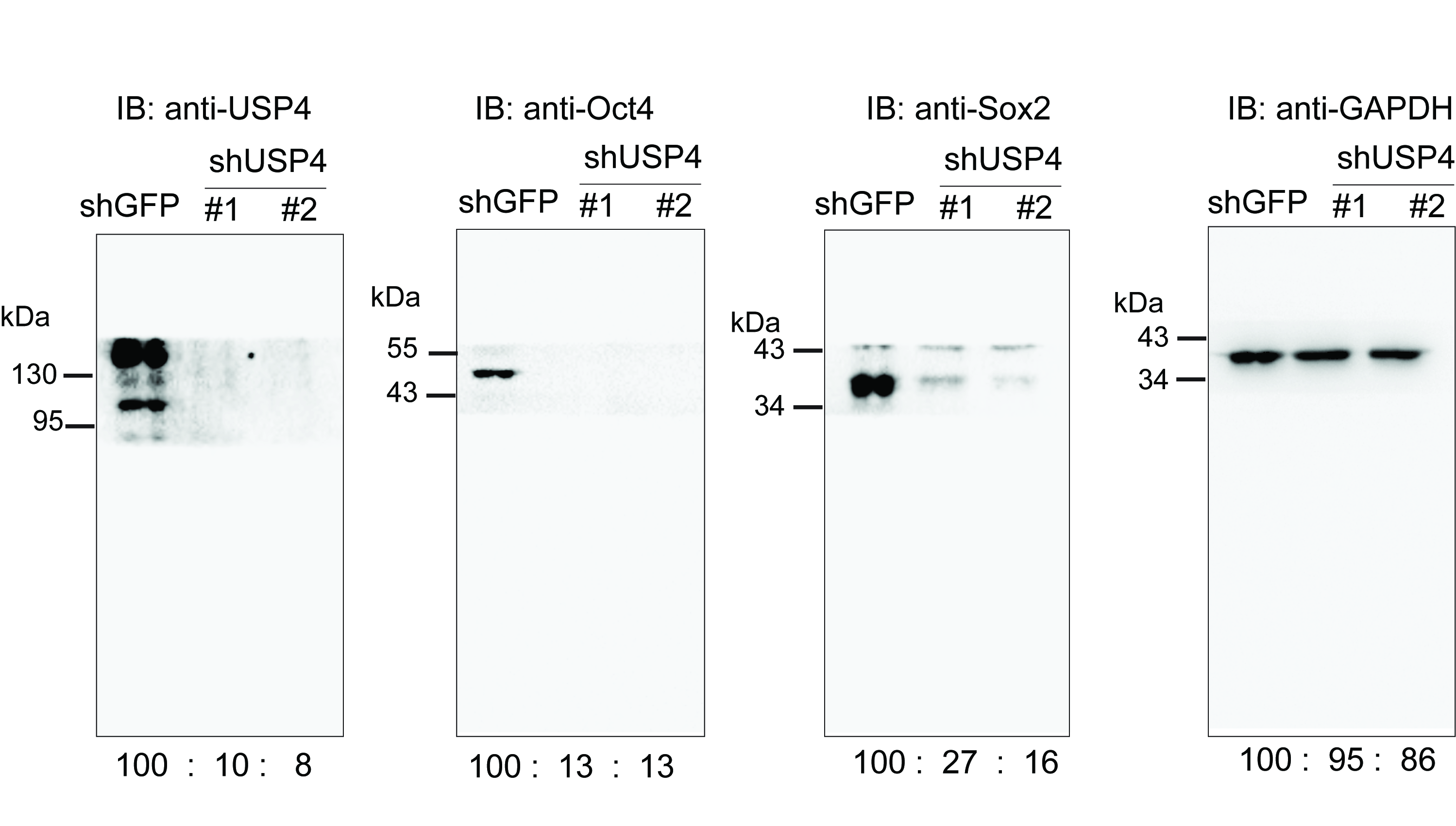

Supplement: Supplementary file 1 [file cancers-12-01582-s001.zip › Suppmentary materials/revised-original-WB-figures/Supplementary-Figure2A.tif]

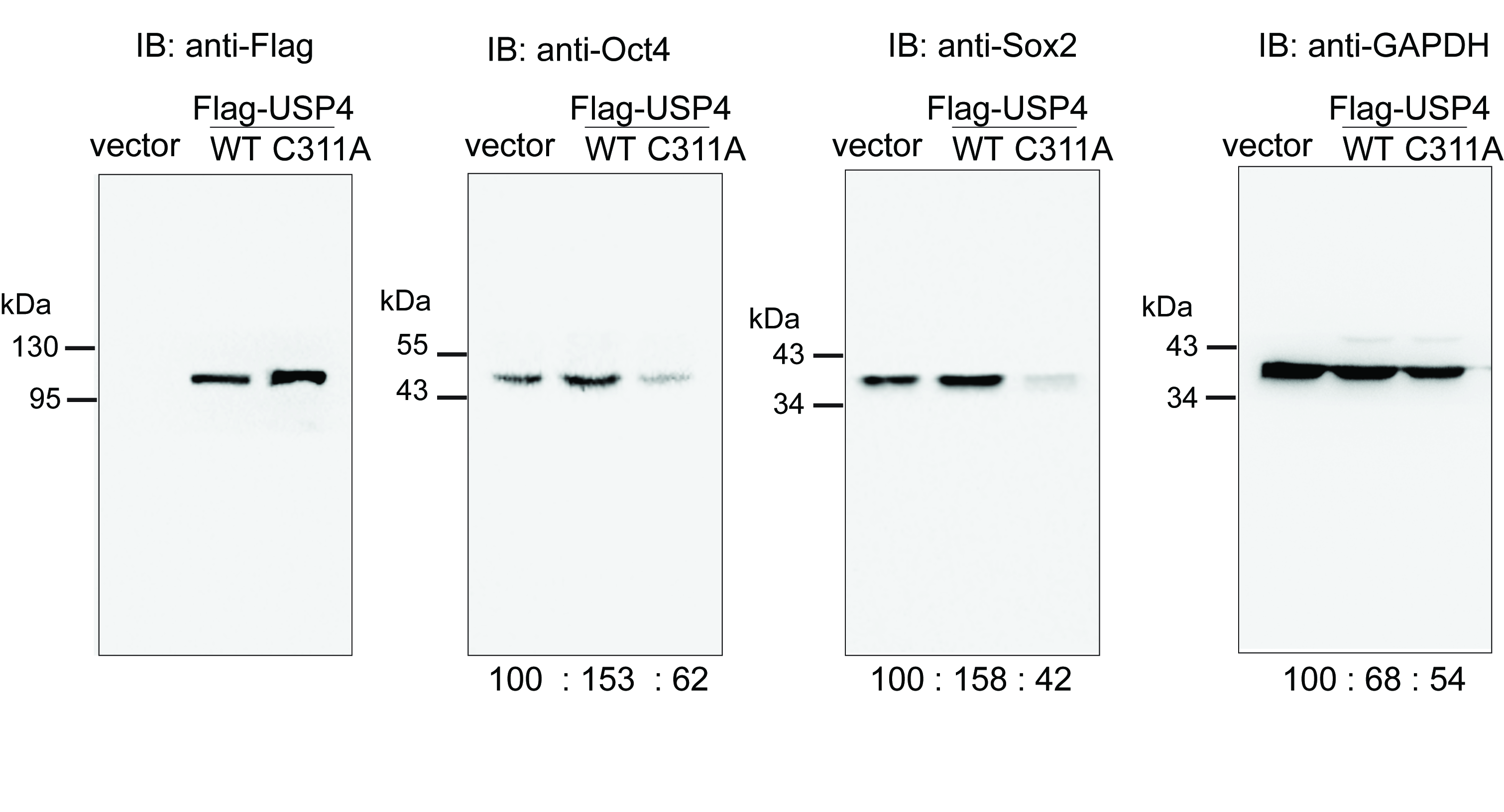

Supplement: Supplementary file 1 [file cancers-12-01582-s001.zip › Suppmentary materials/revised-original-WB-figures/Supplementary-Figure2B.tif]

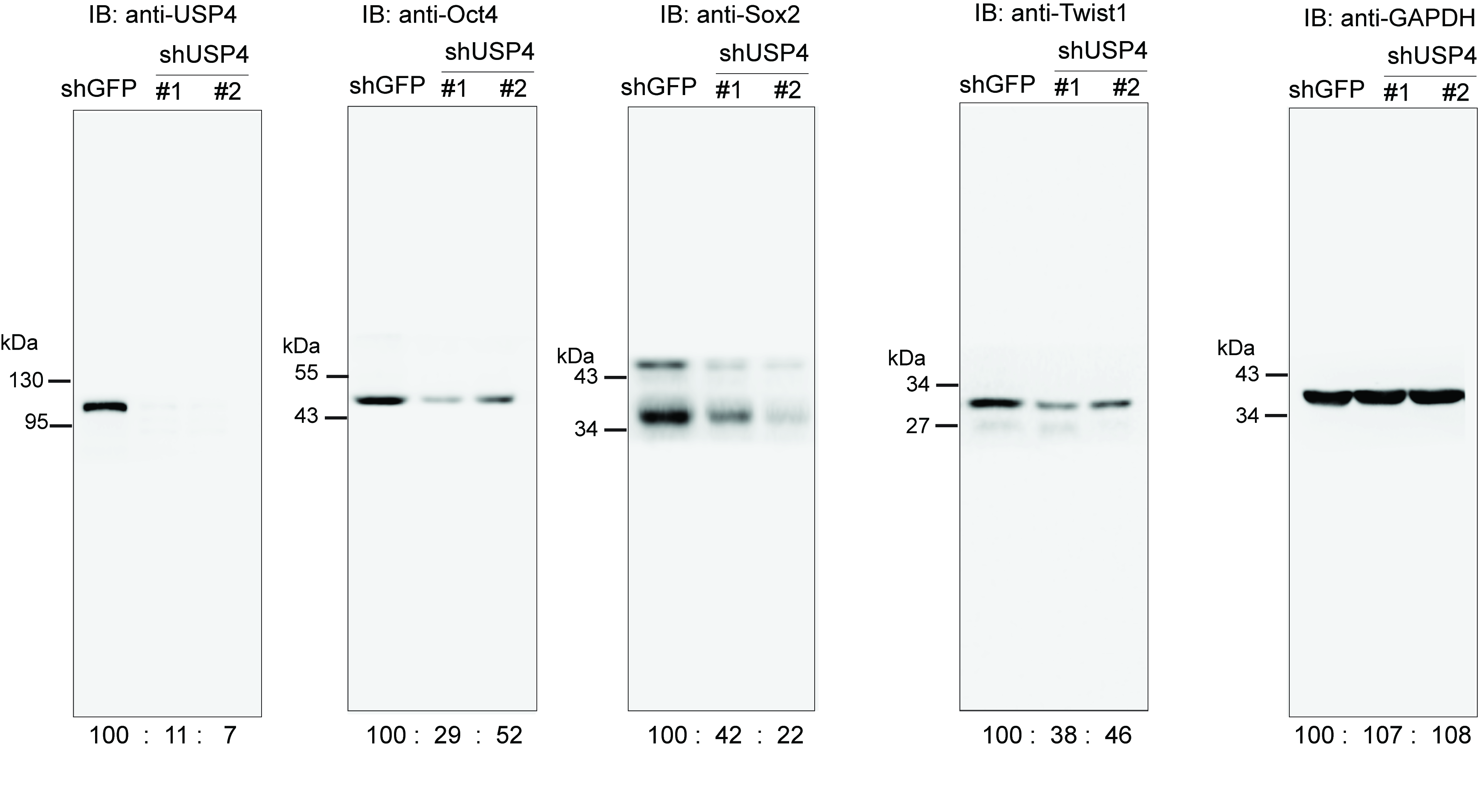

Supplement: Supplementary file 1 [file cancers-12-01582-s001.zip › Suppmentary materials/revised-original-WB-figures/Supplementary-Figure3A.tif]

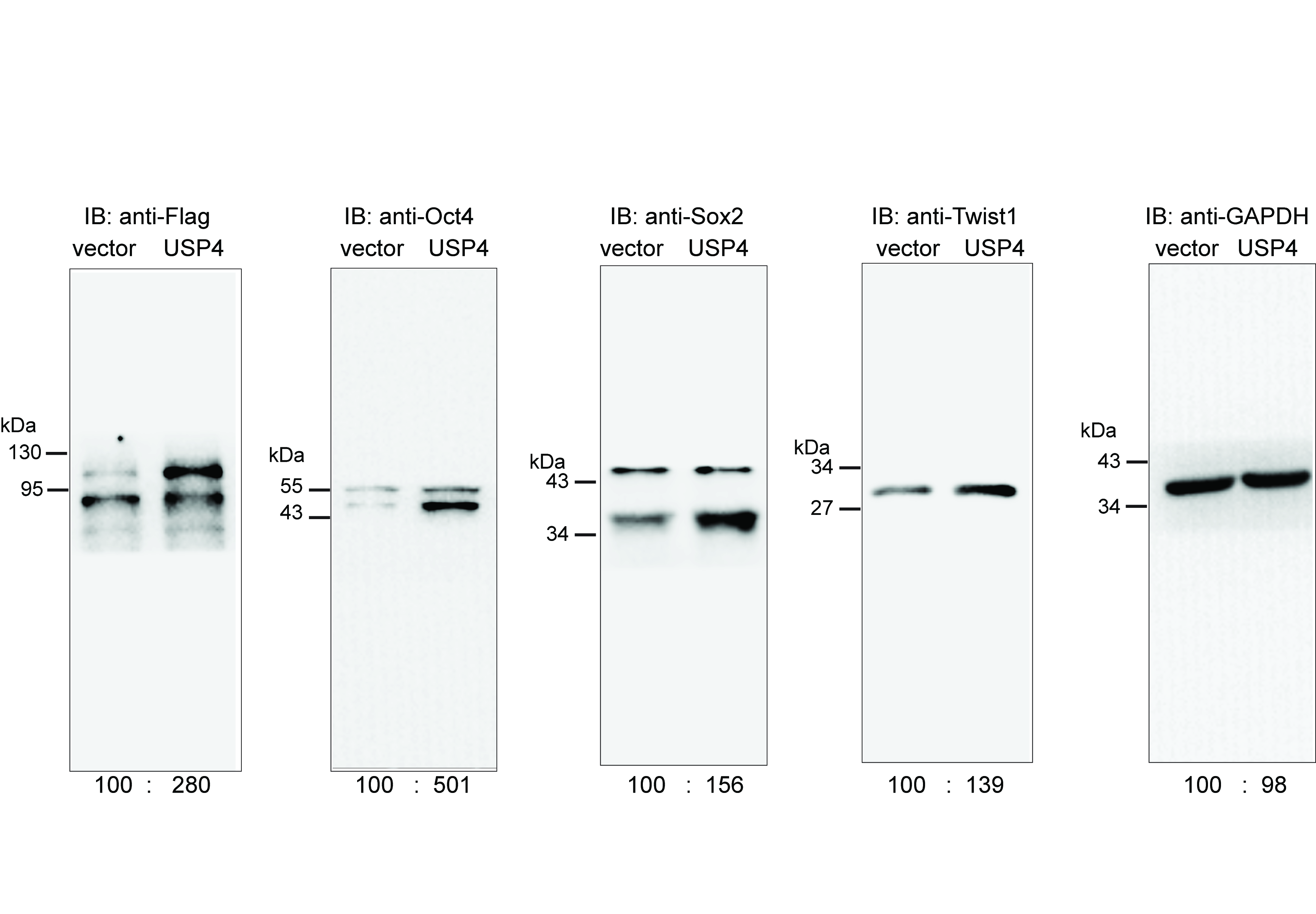

Supplement: Supplementary file 1 [file cancers-12-01582-s001.zip › Suppmentary materials/revised-original-WB-figures/Supplementary-Figure4A.tif]
